# Candidate pathways and genes for prostate cancer: a meta-analysis of gene expression data

**DOI:** 10.1186/1755-8794-2-48

**Published:** 2009-08-04

**Authors:** Ivan P Gorlov, Jinyoung Byun, Olga Y Gorlova, Ana M Aparicio, Eleni Efstathiou, Christopher J Logothetis

**Affiliations:** 1 Department of Genitourinary Medical Oncology, The University of Texas M. D. Anderson Cancer Center, Houston, TX, USA; 2 Department of Epidemiology, The University of Texas M. D. Anderson Cancer Center, Houston, TX, USA

## Abstract

**Backgound:**

The genetic mechanisms of prostate tumorigenesis remain poorly understood, but with the advent of gene expression array capabilities, we can now produce a large amount of data that can be used to explore the molecular and genetic mechanisms of prostate tumorigenesis.

**Methods:**

We conducted a meta-analysis of gene expression data from 18 gene array datasets targeting transition from normal to localized prostate cancer and from localized to metastatic prostate cancer. We functionally annotated the top 500 differentially expressed genes and identified several candidate pathways associated with prostate tumorigeneses.

**Results:**

We found the top differentially expressed genes to be clustered in pathways involving integrin-based cell adhesion: integrin signaling, the actin cytoskeleton, cell death, and cell motility pathways. We also found integrins themselves to be downregulated in the transition from normal prostate tissue to primary localized prostate cancer. Based on the results of this study, we developed a collagen hypothesis of prostate tumorigenesis. According to this hypothesis, the initiating event in prostate tumorigenesis is the age-related decrease in the expression of collagen genes and other genes encoding integrin ligands. This concomitant depletion of integrin ligands leads to the accumulation of ligandless integrin and activation of integrin-associated cell death. To escape integrin-associated death, cells suppress the expression of integrins, which in turn alters the actin cytoskeleton, elevates cell motility and proliferation, and disorganizes prostate histology, contributing to the histologic progression of prostate cancer and its increased metastasizing potential.

**Conclusion:**

The results of this study suggest that prostate tumor progression is associated with the suppression of integrin-based cell adhesion. Suppression of integrin expression driven by integrin-mediated cell death leads to increased cell proliferation and motility and increased tumor malignancy.

## Background

Global profiling of gene expression by microarray technology is an effective tool for studying molecular mechanisms underlying different aspects of carcinogenesis. Unfortunately, the results of the profiling of gene expression are often inconsistent. The discrepancy can be due either to inherent molecular heterogeneity of tumors or to technical artifacts. Meta-analysis was proposed as an approach for identifying a core gene-expression signature reproducible across multiple studies. Several methods of meta-analysis have been suggested [1-5]. One of the recent developments is Bayesian cell mixture modeling, which is applicable to gene as well as protein expression microarrays [3,6-8]. Implementation of these and other methods of meta-analysis identified gene-expression signatures associated with different aspects of tumorigenesis, including prostate tumorigenesis [6,9-13].

The molecular mechanisms of prostate tumorigenesis remain poorly understood [14]. Androgen receptor signaling is critical to prostate cancer development as androgen receptors regulate the proliferation of prostate epithelial cells through several cyclin-dependent kinases [15,16]. Because of the central role of androgen stimulation in prostate tumorigenesis, androgen ablation remains the primary therapy for patients with metastatic disease, yet more effective treatments are desperately needed. There is evidence that other genes can also contribute to prostate tumorigenesis [17-21].

Recent studies have suggested that cell adhesion plays a role in the initiation and progression of prostate cancer. Integrins are cell-surface receptors that interact with extracellular matrix and mediate various intracellular signals. They define cellular shape and motility and also regulate the cell cycle [22-25]. A recent article by Goel et al. [26] provides a review of the expression of integrins in prostate cancer progression with reference to specific integrins. Integrins have been shown to be largely downregulated in prostate cancer development, although some integrins have been found to be upregulated in prostate cancer [22,26,27]. The observed variation in integrin expression may reflect a genetic heterogeneity of prostate tumors: different tumors may exploit different sets of genes to modulate the same functions. On the basis of this working hypothesis, we focused largely on the analysis of functions rather than the analysis of individual genes. The benefit of an analysis at the functional rather than gene level is that the results of such analysis can be more consistent across studies because different tumors may suppress or activate the same function through different genes, making prostate tumors heterogeneous at the gene expression level and much more homogeneous at the pathway level.

First, we identified genes that were differentially expressed at different stages of prostate tumorigenesis and then applied bioinformatics tools to identify the functions associated with such genes and, therefore, with tumor progression. Integrin signaling emerged as the top biologic function modulated in prostate tumorigenesis. Based on the results of our analysis, we propose the "collagen hypothesis" of prostate tumorigenesis, suggesting that the disruption of integrin-based cell adhesion to the extracellular matrix is a driving force for the development of prostate cancer.

## Methods

### Datasets

For the list of the datasets used in this study, please see Additional file 1. Gene expression datasets were retrieved from the Oncomine 3.0 database, accessed in May 2008 [28]. We used 18 datasets in total: 11 datasets for the transition from normal prostate (NP) to nonmetastatic prostate cancer (nMPC) and 7 for the transition from localized (nMPC) to metastatic prostate cancer (MPC). To analyze the genes involved in prostate cancer initiation – transition from normal to prostate intraepithelial neoplasia (PIN) – we used data from a study by Tomlins et al. [29]. Individual *P *values detected in each study were used to compute global *P *values using meta-analysis.

### Data quality control

Combining the results of multiple independent studies allows more robust statistical conclusions than does the analysis of a single dataset. However, including a flawed study in such a meta-analysis could lead to a bias; therefore, the quality of the data must be carefully considered before including a given dataset in the analysis. To ensure reproducibility of the data, we assessed the overlap of significant genes in independent datasets, our rationale being that studies targeting the same phenotype should be expected to detect an overlapping set of differentially expressed genes. We therefore excluded studies wherein the overlap in up/up- or down/down-regulated genes was lower than would be expected in such pairwise comparisons.

We assessed the overlap between significant genes (significant genes were defined by the liberal *P *value of 0.05) that were up- or downregulated in two studies. The results of the analysis were presented using the 2 × 2 data format. For each cell in the 2 × 2 table, we computed the expected number of genes. If N_1,2 _were the number of genes analyzed in both study 1 and study 2, P_u1 _and P_u2 _would be the proportion of genes upregulated in studies 1 and 2, correspondingly. Thus, the expected number of genes upregulated in both studies could be computed as a product of the corresponding proportions and the total number of genes analyzed in both studies. For example, the expected number of genes upregulated in both studies E_u/u _may be computed as E_u/u _= P_u1_P_u2_N_1,2_. A typical example is shown in Table 1. The observed number of genes consistently (in terms of direction, up or down) differentially expressed (up/up or down/down) was much higher than the expected number (χ^2 ^= 6,581.2, df = 1, *P *value extremely low – essentially zero for this statistic). Gene expression data from two independent studies [30] and [31] were used in this analysis. The observed numbers of genes not consistent in their expression (up/down and down/up) were similar to that expected (χ^2 ^= 2.1, df = 1, *P *= 0.15), suggesting that genes upregulated in one study but downregulated in another were likely to be false positives.

**Table 1:** Observed and expected (in parentheses) numbers of significant genes (*P *≤ 0.05) with the same or opposite direction (up/down) of differences in gene expression in studies [30] and [31]*

		Data from [31]	
		
		DOWN	UP
Data from [30]	DOWN	**905 (165)**	116 (106)
	
	UP	76 (86)	**454 (50)**

* Both studies compared gene expression in NP vs. that in primary nMPC tumors.

If the ratio of the observed to the expected number of genes were to be close to 1 for all cells in a 2 × 2 table, it would suggest that at least one study was severely flawed and should be excluded from analysis. In a comparison of just two studies, it would be impossible to say which one should be excluded. However, if all but one among multiple (i.e., six) datasets showed a considerable overlap, we could conclude that the inconsistent study should be excluded. On the basis of this approach, we excluded two studies from the original list of datasets.

### False-discovery rate

**Figure 1:**
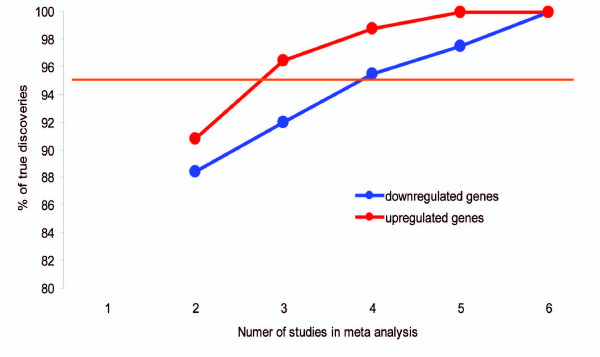
**Dependence of the percentage of true discoveries on the number of studies in meta-analysis**. The percentage is equal or higher than 95% when 4 or more independent studies were included in meta analysis.

We computed the expected number of significant genes based on the strict null hypothesis, which assumes that all findings are technical or statistical artifacts and that a random set of genes is detected as significant in each individual study. The excess of the observed over the expected number of genes would then be due to the presence of true statistical positives. The proportion of true positives among all significant genes can be computed as *P*_*t *_= (*Nobs *- *Nexp*)/*Nobs*. We estimated *P*_*t *_across different numbers of independent studies used in meta-analysis. We found that combining six or more independent studies in a meta-analysis provided statistically robust detection of true positives. Figure 1 shows the dependence of the proportion of true positives on the number of studies used in meta-analysis, with the combination of four independent studies reducing the probability of false positives to lower than 0.05. If six studies were included, the probability of false positives would be lower than 0.01.

Note that our approach for the assessment of the proportion of true positives is different from the estimate of false discovery rate (FDR) that is based on the Bonferroni correction and computed as a product of the type one error and the total number of tests [32]. The FDR approach takes into account the possibility that false significant findings could result from multiple testing, whereas an inflation of statistics resulting from technical artifacts or poor study design would be treated as a true signal. Our approach was more conservative, allowing for the exclusion of false positives resulting from technical artifacts and flaws in study design.

### Meta-analysis

We used an extension of Stouffer's method [33] to estimate the overall significance based on the significance of the individual tests. This approach is based on estimating the standard normal deviation *Z *and is similar to the approach recently proposed by Ochsner et al. [1]. Selection of the method of meta-analysis for our study was dictated by the available data: for the majority of the datasets, the raw gene-expression data were not available, while *t *tests and corresponding *P *values were easily accessible for individual probes.

Individual probability *P *is first converted into a Z score and the Z scores summed up across studies. This sum is divided by the square root of the number of tests (k). The k was the number of datasets where the specific gene was assessed: maximum 11 for the NP>nMPC and 7 for the nMPC>MPC transition. The sum of normal deviates is itself a normal deviate and can be backtransformed into an overall *P*; the probability level associated with the sum of Z yields an overall level of significance. The complete procedure takes the following steps: **(1) **P_i _→ Z_i_; **(2) **Z_(overall) _= ΣZ_i_/; **(3) **Z_(overall) _→ P_(overall)_. The advantage of this method lies in the increased power of the overall comparison. If, for example, several tests consistently favored the research question but failed to reach the level of significance due to small sample size, the overall test would more easily become significant because of the pooled sample size being much larger than its components.

For each gene, we computed the overall global *P *values based on individual *P *values from independent studies. We then ranked all genes according to the *P *value from smallest to largest. We used the top-ranked genes to analyze their clustering by pathway, molecular, and cellular functions.

### Functional annotation

For functional annotation, we applied Ingenuity Pathways Analysis (Ingenuity Systems, ) and the Database for Annotation, Visualization, and Integrated Discovery (DAVID) [34]. We analyzed the data at three levels: that of (i) gene, (ii) pathway, and (iii) biologic function. Ingenuity and DAVID use similar approaches for functional annotation of candidate genes, looking at the distribution of the top-ranked genes by pathways and/or gene ontology categories, testing the null hypothesis that genes are randomly distributed across pathways and biologic functions. *P *values characterize the statistical evidence for the clustering of the genes by pathways or functional categories; the lower the *P *value, the stronger the statistical evidence that the top-ranking genes belong to a specific pathway or functional category. In this study, we used the classification of pathways according to the Kyoto Encyclopedia of Genes and Genomes (KEGG) [35]. The KEGG provides a collection of manually drawn pathway maps representing current knowledge about the molecular interactions for metabolism, processing of genetic and environmental information, cellular processes, and human diseases [36].

To decide how many top-ranked genes to include in functional annotation analysis, we analyzed the dependence of the number of significant pathways and functional categories on the number of top genes (see section "How many genes to use for an assessment of functional clustering" in Additional file 2 for details of the analysis). On the basis of the results of the analysis, we decided to use the top 500 genes for functional annotation.

## Results

### Functional annotation at the gene level

**Figure 2:**
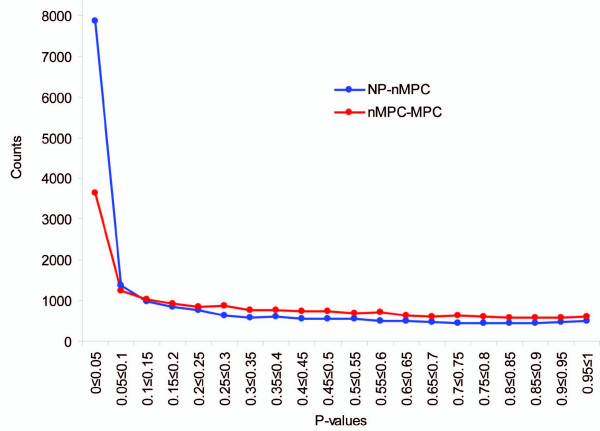
**The distributions of the *P *values for the transitions from normal prostate to primary nonmetastatic prostate cancer (NP – nMPC) and primary nonmetastatic to metastatic prostate cancer (nMPC – MPC)**.

Because we analyzed many genes, it is possible that some significant findings could be the result of multiple testing. Figure 2 shows the distribution of *P *values for the genes tested in the transition phases from NP to nMPC (NP-nMPC) and from nMPC to MPC (nMPC-MPC). For both transitions, a significant excess of low *P *values was observed, suggesting the presence of true positives.

#### Functional annotation at the pathway level

##### The NP-nMPC transition

The top 500 genes, accounting for about 2% of all known genes in the human genome, identified by meta-analysis of the gene expression data were used for functional annotation. The list of the 500 most significant genes in the NP-nMPC transition is shown in Additional file 3. There were approximately equal numbers of up- and downregulated genes: 231 and 269 genes, respectively.

Analysis of the distribution of genes by pathways defined by the KEGG revealed clustering of the differentially expressed genes in focal adhesion pathway. We found that 21 genes fell into focal adhesion pathways: *RAP1A, PRKCA, COL6A1, IGF1, ITGA3, PARVA, CAV2, PIK3R1, FLNA, MYL9, FLNC, PPP1R12A, THBS4, PRKCB1, CAV1, ACTN1, COL4A6, MET, ITGA2, VCL*, and *MYLK*. A schematic of the focal adhesion pathway can be found in Additional file 4. Interestingly, all genes except *THBS4 *were downregulated. The difference between the observed and the expected (based on the overall proportion of downregulated genes) number of downregulated genes in this group was significant (χ^2 ^= 14.5, df = 1; *P *< 0.0001).

Functional annotation of the top genes by Ingenuity produced similar results (Table 2, see additional file 5 for the list of the genes in each pathway). The actin cytoskeletal signaling pathway was one of the top pathways enriched by the differentially expressed genes. Cell-adhesion pathways, namely tight junction and integrin signaling, were also significant.

**Table 2:** Canonical pathways significant in the transition from normal prostate to localized prostate cancer (functional annotation by Ingenuity)*

Pathway	-Log (P)
Calcium signaling	4.62
NRF2-mediated oxidative stress response	4.05
Actin cytoskeleton signaling	3.31
Tight junction signaling	3.19
Synaptic long term potentiation	3.05
Hepatic fibrosis/hepatic stellate cell activation	2.99
Integrin signaling	2.58
EGF signaling	2.45
GM-CSF signaling	2.38
Regulation of actin-based motility by Rho	2.12
Wnt/beta-catenin signaling	2.04
PDGF signaling	2.04
Xenobiotic metabolism signaling	1.98
Chemokine signaling	1.94
Arginine and proline metabolism	1.94
Cell cycle: G1/S checkpoint regulation	1.86
ERK/MAPK signaling	1.79
cAMP-mediated signaling	1.77
VEGF signaling	1.65
Leukocyte extravasation signaling	1.61
Neuregulin signaling	1.60
Nitric oxide signaling in the cardiovascular system	1.54
Glutathione metabolism	1.54
LPS/IL-1 mediated inhibition of RXR function	1.52
Purine metabolism	1.41
Fatty acid biosynthesis	1.34
Aryl hydrocarbon receptor signaling	1.34
Glucocorticoid receptor signaling	1.34

*Lists of the significant genes in each pathway are shown in Additional file 5.

##### The nMPC-MPC transition

Additional file 6 shows the top 500 genes differentially expressed in the transition from nMPC to MPC. Functional annotation using the KEGG database identified focal adhesion as a top pathway enriched by the differentially expressed genes. We found 17 focal adhesion genes differentially expressed in nMPC vs. MPC: *ILK, RAP1A, PARVB, AKT3, JUN, FLNA, GRB2, MYL9, FLNC, THBS2, PPP1R12A, THBS4, FYN, COL4A6, VCL, MYLK*, and *PPP1CB*. The majority (13 of 17) were downregulated. Nine of the 17 genes – *COL4A6, FLNA, FLNC, MYL9, MYLK, PPP1R12A, RAP1A, THBS4*, and *VCL *– were also differentially expressed in the NP-nMPC transition. All of them except *THSB4 *were downregulated in both transitions, suggesting similar modulation of the pathway.

Functional annotation by Ingenuity identified 17 significant canonical pathways enriched by the genes differentially expressed between nMPC and MPC (Table 3, see additional file 7 for the list of the genes in each pathway). The top five canonical pathways included those of tight junction, IGF-1, integrin, and ERK/MAPK signaling, as well as that of regulation of actin-based cell motility by Rho. Genes involved in the cell adhesion, tight junction signaling, and integrin signaling pathways again showed clear tendencies to be downregulated. For the tight junction signaling, 13 of 14 genes were downregulated and, for integrin signaling, 11 of 14 genes were downregulated.

**Table 3:** Canonical pathways significant in nonmetastatic prostate cancer to metastatic prostate cancer transition identified by Ingenuity*

Pathway	-Log(P)
Tight junction signaling	3.37
IGF-1 signaling	2.68
Integrin signaling	2.33
ERK/MAPK signaling	2.32
Regulation of actin-based motility by Rho	2.22
Hepatic fibrosis/hepatic stellate cell activation	2.14
Cardiac beta^2^-adrenergic signaling	1.92
EGF signaling	1.86
Actin cytoskeleton signaling	1.84
PPARα/RXRα activation	1.72
IL-4 signaling	1.71
Nitric oxide signaling in the cardiovascular system	1.62
Hypoxia signaling in the cardiovascular system	1.62
Antigen presentation pathway	1.51
Glucocorticoid receptor signaling	1.46
Glycolysis/gluconeogenesis	1.40
TGF-beta signaling	1.36

*Lists of the significant genes in each pathway are shown in Additional file 7.

#### Combined analysis of genes involved in NP-nMPC and nMPC-MPC transitions

The considerable overlap between pathways involved in the NP-nMPC and nMPC-MPC transitions suggests that the same pathways might be involved in both transitions. Therefore, we conducted a pooled meta-analysis of NP-nMPC and nMPC-MPC transitions. The more benign phenotype always was used as the reference: NP for NP-nMPC and nMPC for nMPC-MPC.

Table 4 provides a list of significant canonical pathways identified in the combined analysis (see additional file 8 for the list of the genes in each pathway). The top canonical pathways were integrin, actin cytoskeleton, tight junction, and chemokine signaling.

**Table 4:** Canonical pathways identified in the combined analysis of genes differentially expressed between the transitions from normal prostate to primary nonmetastatic prostate cancer and from primary nonmetastatic to metastatic prostate cancer*

Pathway	-Log(P)
Integrin signaling	4.42
Hepatic fibrosis/hepatic stellate cell activation	3.50
Actin cytoskeleton signaling	3.27
Tight junction signaling	3.16
Chemokine signaling	3.10
Calcium signaling	2.60
IGF-1 signaling	2.54
Aryl hydrocarbon receptor signaling	2.10
Regulation of actin-based motility by Rho	2.10
Nitric oxide signaling in the cardiovascular system	2.05
Beta-alanine metabolism	2.03
Wnt/β-catenin signaling	1.63
VEGF signaling	1.63
p53 signaling	1.48
NRF2-mediated oxidative stress response	1.43
Arginine and proline metabolism	1.42
Cardiac β-adrenergic signaling	1.38
cAMP-mediated signaling	1.38
Cell cycle: G2/M DNA damage checkpoint regulation	1.33

*Lists of the genes for each pathway are shown in Additional file 8.

### The downregulation of integrins and integrin ligands

We found that several adhesion pathways, including focal adhesion, integrin signaling, and tight junction signaling, were significantly associated with prostate tumorigenesis. Therefore, we decided to look at the expression of cell adhesion genes in more detail. We stratified cell adhesion genes into five categories: cadherins, immunoglobulin-like adhesion genes (ICAMs), integrins, selectins, and tight junction genes. We found that integrins tended to be downregulated in prostate nMPC compared with NP (see Additional file 9). Of 24 integrins and the proteins directly binding them, 20 genes, or 83%, were downregulated, and only four were upregulated, compared with the expected 46% based on the overall analysis of significant genes at *P *< 0.05 (χ^2 ^= 11.4, df = 1, *P *= 0.0003). The excess of downregulated integrins was even more striking when only integrins themselves were considered: all except *ITGAX *were downregulated. Other types of cell adhesion genes (cadherins, ICAMs, selectins, and tight junction genes) also exhibited predominant (although less profound) downregulation (see Additional file 9).

Integrin ligands also showed a tendency to be downregulated. In the NP-nMPC transition, 30 of 38 differentially expressed integrin ligands were downregulated (Additional file 10). In the nMPC-MPC transition, the number of downregulated integrin ligands was similar to the number of upregulated integrin ligands: 32 and 28, respectively. The major sources of integrin ligands in prostate tissue are fibroblasts, although some investigators suggest that epithelial cells can also produce integrin ligands [37]. We tried to distinguish between the two sources of integrin ligands by separately analyzing grossly dissected and laser-capture microdissected tumor cells. We found no collagen, fibrinogen, or laminin genes among the top 500 genes in the two studies that analyzed gene expression in individually dissected tumor cells [29,38]. In the studies that assessed gene expression in a mixture of tumor cells and fibroblasts, we found three differentially expressed collagen genes: *COL15A1, COL4A6*, and *COL4A5*. All three were significantly downregulated; the corresponding *P *values were 7.8E-12, 7.0E-11, and 1.1E-9. These results suggest that differences in expression of extracellular matrix proteins are likely due to differences in gene expression in fibroblasts.

### Early-stage prostate tumorigenesis: NP vs. prostatic intraepithelial neoplasia (PIN)

We also looked specifically at the expression of integrins at the initial stage of prostate tumorigenesis – the transition from NP to PIN – using gene expression data from Tomlins et al. [29]. Ten of 22 integrins analyzed in the study were differentially expressed between NP and PIN. Seven integrins (*ITGA3, ITGA4, ITGA8, ITGB4, ITGA2, ITGAM*, and *ITGB3BP*) were downregulated, with *P *values of 8.2E-5, 0.005, 0.008, 0.01, 0.03, 0.03, and 0.04, respectively. Four genes (*ITGAV, ITGB5, ITGAL*, and *ITGB3BP*) were upregulated in PIN compared with NP, with *P *values of 0.001, 0.0006, 0.009, and 0.02, respectively. When we looked at the expression of integrin ligands, we found that 21 genes (*COL5A3, COL6A1, LAMB2, COL11A2, COL17A1, FN1, LAMA3, COL7A1, COL3A1, COL6A3, COL4A2, LAMB1, COL23A1, COL13A1, COL1A2, COL16A1, COL20A1, COL9A1, LAMA2, COL4A5*, and *COL15A1*) were differentially expressed between NP and PIN. All ligands except *LAMB1 *were downregulated, suggesting that suppression of integrin ligands plays a role in the initiation of prostate cancer.

Additionally, we compared the expression of 69 cell-adhesion molecules: cadherins, integrins, selectins, ICAMs, and the genes involved in the formation of tight junctions between stromal and epithelial cells. We used the data from the study conducted by Tomlins et al. [29]. The list of the cell-adhesion genes was based on the genes from two cell-adhesion databases [39,40]. Of 69 cell-adhesion genes, 63 did not show a difference in the gene expression level between stromal and epithelial cells. Of six differentially expressed genes, three – *CDH1, ITGB5*, and *TJP3 *– were downregulated and three – *ICAM1, ITGA8*, and *ITGB5 *– were upregulated in the stroma relative to the level in the epithelium.

### Overlap between NP-nMPC and nMPC-MPC transitions at the gene, pathway, and function levels

We found that 77 or 15% of the top 500 genes involved in the NP-nMPC and nMPC-MPC transitions were the same. This overlap was much stronger than would be expected purely by chance. Indeed, 17,859 genes were assessed in both the NP-nMPC and nMPC-MPC analyses, making the proportion of the top 500 (500/17859) to be 0.03. The probability that the same gene would be found among the top 500 in both studies would thus be 0.03*0.03 = 0.0009 (about 0.1%), or 0.5 genes compared with the 77 found in our analysis.

At the pathway level, 10 of 18 pathways, or 56%, were the same in the NP-nMPC and nMPC-MPC transitions. Overlap at the functional level was even stronger: 18 of 21, or 86%, of functional categories significant in the transition from NP to primary nMPC were also significant for the transition from nMPC to MPC.

### Correlation between gene expression and Gleason score

We looked at the correlation between gene expression and Gleason score by using data from four studies [41-44]. We found that expression of *ITGA5 *and *ITGAL *were significantly negatively correlated with Gleason score, with corresponding *P *values of 0.003 and 0.02. We also considered the correlation of the expression of integrin ligands (that are largely components of the extracellular matrix) with the Gleason score. We found that the expression levels of seven integrin ligands (*COL4A6, COL13A1, FGB, COL19A1, COL18A1, COL14A1*, and *COL11A2*) were negatively correlated with the Gleason score, suggesting that the suppression of integrins and/or their ligands is associated with more advanced tumor grade.

## Discussion

Microarray technology allows the simultaneous assessment of all (or most) of the genes in the human genome, making it the prime method for this genome-wide study of gene expression. Numerous studies of this sort have been conducted, providing a basis for a meta-analysis of gene expression data [29,45,46]. Unfortunately, microarray-based analysis of gene expression is sensitive to numerous technical and statistical biases, making the results of any individual analysis unreliable. A meta-analysis may generate a more robust and reproducible list of the genes differently expressed during prostate tumorigenesis. Several meta-analyses of prostate tumorigenesis have been undertaken. To our best knowledge, the first study was conducted by Ghosh et al. [47]. The meta-analysis combined the results of four independent studies. The authors identified several metabolic pathways, including purin metabolism and oxidative phosphorylation. A meta-analysis of seven microarray datasets was used to evaluate the role of the TGF-beta pathway in prostate tumorigenesis [48]. In another meta-analysis, datasets from a mouse model were used to assess the role of integrin alpha7 in prostate cancer progression [49]. The results of that study suggest that integrin alpha7 plays a role in metastasizing and cancer free survival. Finally, a recent meta-analysis of four independent datasets profiling the gene expression in normal prostate versus tumor demonstrated involvement of the Wnt and p53 signaling pathways in prostate tumorigenesis [50]. In our analysis, TGF-beta, Wnt, and p53 signaling were also among the top pathways associated with prostate tumor progression.

In this study, we found cell adhesion, death, proliferation, and motility to be the top functions having differential expression in both NP-nMPC and nMPC-MPC transitions. All these functions are mechanistically connected with each other. Integrin-based cell adhesion provides direct mechanical links between extracellular matrix and actin cytoskeleton [51,52]. Deregulation of actin cytoskeletons caused by weakened cell adhesion directly modulates cell motility, proliferation, and death, providing a molecular basis for the histologic changes in prostate tumorigenesis.

We found that prostate tumor progression is associated with suppressed expression of integrins. Published evidence also supports the involvement of integrins in prostate tumorigenesis. A recent paper by Goel et al. [26] provides an excellent review of studies on the role of integrins in prostate cancer development. Consistent with our results, most of the integrins were reported to be downregulated in prostate cancer in that article, as shown by both immunohistochemistry and assessments of gene expression on the mRNA level (see [26] Table 1, and [53,54]). Moreover, transfection-mediated expression of β_1 _integrin, which is downregulated in prostate cancer, induces cell adhesion to laminin and prevents tumor growth [55]. Further, it was demonstrated that induced expression of ITGA7 suppresses growth of prostate tumors in mice [56].

Weakening of integrin-mediated cell adhesion to the extracellular matrix may initiate and drive prostate tumorigenesis. Recent studies by others have demonstrated that binding integrins to integrin ligands is crucial for cell survival in vivo [57-60]. The presence of integrins not bound to ligands induces apoptosis through caspase 8-integrin-mediated cell death [59,60]. Cells can escape integrin-mediated death if they suppress the expression of ligandless integrins [59]. Suppression of caspase 8 apoptotic signaling coupled with activation of several prosurvival pathways was suggested as a possible mechanism of overcoming integrin-mediated cell death [58,59].

These data, in combination with the results of our study, provide a basis for the collagen hypothesis of prostate tumorigenesis. We believe that the primary event in prostate tumorigenesis is decreased expression of collagen genes, a normal physiologic process associated with aging [61]. Age-associated depletion of collagens leads to the accumulation of ligandless integrins and induction of integrin-associated cell death, as noted. Integrin-associated death is a driving force behind prostate tumorigenesis, as cells attempt to escape this by suppression of integrin expression. Suppression of integrins, in turn, elevates malignant potential by elevating cell motility and proliferation and leads to disorganized prostate histologic features.

Integrin-associated cell death may also provide a molecular mechanism underlying the prostatic atrophy frequently observed in elderly men. It has been hypothesized that some of these lesions can be precursors to prostate tumor because they are frequently colocalized with carcinoma, and their gene expression pattern resembles that of tumor cells [62,63].

Several features of this collagen hypothesis make it unique among hypotheses of prostate tumorigenesis [64]. First, the initial impetus comes from the prostate cell environment rather than from the epithelial cells themselves. Second, the environment changes are due not to random mutations but rather to a normal physiologic process associated with aging: depletion of collagens and other integrin ligands. Somatic mutations or epigenetic changes may play a role in integrin suppression, allowing cells to escape integrin-associated death. The development of prostate cancer is thus a physiologic response to the depletion of integrin ligands associated with this aging process. The collagen hypothesis provides hope that researchers could prevent or slow the development of prostate cancer by preventing age-associated collagen depletion. This hypothesis also suggests that activation of integrin expression could reattach the cells to the extracellular matrix, reversing their cancerous phenotype.

What is the place of integrin signaling pathways in the framework of known genes affecting prostate cancer development? We believe that age-associated suppression of integrin-dependent cell adhesion provides a background for prostate cancer development by increasing the effects of other genes involved in cancer development. It has been demonstrated that cell adhesion can modulate the effects of androgen signaling [65]. Several other genes involved in prostate cancer development show strong dependence on cell adhesion [66-70]. This suggests that the suppression of extracellular matrix cell adhesion could enhance the effect of other genetic modulators of prostate cancer development.

A limitation of the approach used in our study is that microarray-based assessment of gene expression does not allow estimation of the expression of splice isoforms. It is known that up to 70% of genes in the human genome are alternatively spliced [71]. In some cases, alternatively spliced integrins have been demonstrated to have distinct functions and expression patterns [72]. The gene expression data used in this analysis provide an integrative measure of gene expression; a more detailed assessment of gene expression is needed for candidate genes. Another limitation of our study is that the majority of the samples was obtained by surgical dissection of tumor tissue and therefore represent a mix of different cell types. For some genes, drastic differences in the expression level between cell types have been reported [73]. Differences in the expression level between different cell types can contribute in the observed difference in gene expression at the tissue level. To address this, we separately conducted functional annotation of the top 500 genes identified in the studies with samples obtained through laser capture microdissection of tumor cells [29,46]. Although only about one third of all known cell-adhesion genes were analyzed in these studies, functional annotation identified cell adhesion and cytoskeleton as the top pathways, suggesting their involvement in prostate carcinogenesis.

As a next step, we plan to analyze integrin signaling pathway genes from independent clinically confirmed prostate tumors at different stages of tumor progression. We also plan to analyze stromal and tumor cells separately, which will allow the estimation of the expression of integrins and integrin ligands separately in tumor and the surrounding stroma.

## Conclusion

The results of this study suggest that prostate tumor progression is associated with the suppression of integrin-based cell adhesion. Suppression of integrin expression driven by integrin-mediated cell death leads to increased cell proliferation and motility and increased tumor malignancy.

## Competing interests

The authors declare that they have no competing interests.

## Authors' contributions

IPG and CJL conceived the study and participated in its design. IPG and JYB were responsible for the data analysis. IPG, OYG, EE, AMA and CJL participated in study design, discussion, and in the manuscript writing. All authors read and approved the final manuscript.

## Pre-publication history

The pre-publication history for this paper can be accessed here:



## Supplementary Material

Additional file 1 **Description of datasets used for the meta-analysis**. The data provided represent a brief description of the used datasets. Click here for file

Additional file 2 **Description of how many genes to use for an assessment of functional clustering**. Text representing discussion of many genes to use for an assessment of functional clustering. Click here for file

Additional file 3 **List of the top 500 genes differentially expressed between normal prostate and localized prostate tumor – NP-nMPC transition**. The data provided represent the list of the top 500 genes differentially expressed between normal prostate and localized prostate tumor. Click here for file

Additional file 4 **Schematics of cell adhesion pathway (according to the Kyoto Encyclopedia of Genes and Genomes)**. Picture representing cell adhesion pathway. Click here for file

Additional file 5 **List of genes for Table **2** "Canonical pathways significant in the transition from normal prostate to localized prostate cancer (functional annotation by Ingenuity software)"**. The data provided represent the list of genes for Table 2. Click here for file

Additional file 6 **List of the top 500 differentially expressed genes in the transition from localized to metastatic prostate cancer – nMPC-MPC transition**. The data provided represent the list of the top 500 differentially expressed genes in the transition from localized to metastatic prostate cancer. Click here for file

Additional file 7 **List of genes for Table **3** "Canonical significance in nonmetastatic prostate cancer to metastatic prostate cancer (nMPC-MPC) transition pathways identified by Ingenuity software"**. The data provided represent the list of genes for Table 3. Click here for file

Additional file 8 **List of genes for Table **4** "Canonical pathways identified in the combined analysis of genes differentially expressed between the transitions from normal prostate to primary nonmetastatic prostate cancer (NP-nMPC) and primary nonmetatatic to metastatic prostate cancer (nMPC-MPC)"**. The data provided represent the list of genes for Table 4. Click here for file

Additional file 9 **Changes in gene expression of different cell adhesion molecule in NP-nMPC transition**. The data provided represent changes in gene expression of different cell adhesion molecule in NP-nMPC transition. Click here for file

Additional file 10 **Changes in expression of Integrin ligands in the transition from normal prostate to localized nonmetastatic prostate cancer – NP-nMPC transition**. The data provided represent changes in expression of Integrin ligands in the transition from normal prostate to localized nonmetastatic prostate cancer. Click here for file
