# Supplementary material for: Candidate pathways and genes for prostate cancer: a meta-analysis of gene expression data
Source: BMC Med Genomics. 2009 Aug 4;2:48. doi: 10.1186/1755-8794-2-48 (PMC2731785; doi:10.1186/1755-8794-2-48)
Supplement: Additional file 1 — Description of datasets used for the meta-analysis. The data provided represent a brief description of the used datasets. [file 1755-8794-2-48-S1.doc]

Additional File 1.

A short description of datasets used for the meta analysis.

| Name | PMID | Type of specimen | Sample Description  Class1:  Class2: | Total Number of Genes |
| --- | --- | --- | --- | --- |
| Dhanasekaran_Prostate | 11518967 | Surgical dissection | Normal Prostate (22)  Primary Prostate Cancer(59) | 9956 |
| Dhanasekaran_Prostate_2 | 15548588 | Surgical dissection | Normal Adjacent Prostate(12)  Prostate Cancer(25) | 19650 |
| Holzbeierlein_Prostate | 14695335 | Surgical dissection | Normal Prostate(4)  Prostate Cancer(23) | 5854 |
| Lapointe_Prostate | 14711987 | Surgical dissection | Normal Prostate(41)  Prostate Carcinoma(62) | 19116 |
| Luo_Prostate | 11406537 | Surgical dissection | Benign Hyperplasia(9)  Prostate Carcinoma(16) | 6500 |
| Nanni_Prostate | 16513839 | Surgical dissection | Normal Prostate(3)  Prostate Carcinoma(22) | 22283 |
| Tomlins_Prostate | 17173048 | Laser capture | Benign Prostate(22)  Prostate Carcinoma(30) | 19355 |
| Vanaja_Prostate | 12873976 | Surgical dissection | Normal Prostate(8)  Prostate Adenocarcinoma (27) | 44928 |
| Varambally_Prostate | 16286247 | Surgical dissection | Benign Prostate(6)  Prostate Carcinoma(7) | 54675 |
| Welsh_Prostate | 11507037 | Surgical dissection | Normal Prostate(9)  Prostate Carcinoma(25) | 11138 |
| Yu_Prostate | 15254046 | Surgical dissection | Normal Prostate(23)  Prostate Carcinoma(64) | 12625 |
| Dhanasekaran_Prostate | 11518967 | Surgical dissection | Primary Prostate Cancer (59)  Metastatic Prostate Cancer (20) | 9935 |
| Dhanasekaran_Prostate_2 | 15548588 | Surgical dissection | Prostate Cancer (25)  Metastatic Prostate Cancer (6) | 18502 |
| Tomlins_Prostate | 17173048 | Laser capture | Prostate Carcinoma (30)  Metastatic Prostate Cancer (19) | 19337 |
| Yu_Prostate | 15254046 | Surgical dissection | Prostate Carcinoma (64)  Metastatic Prostate Cancer (25) | 12625 |
| Vanaja_Prostate | 12873976 | Surgical dissection | Prostate Adenocarcinoma (27)  Metastatic Prostate Cancer (5) | 44928 |
| Holzbeierlein_Prostate | 14695335 | Surgical dissection | Prostate Cancer (23)  Metastatic Prostate Cancer (9) | 6475 |
| LaTulippe_Prostate | 12154061 | Manual cell dissection | Prostate Carcinoma (23)  Metastatic Prostate Cancer (9) | 12600 |
