# Supplementary material for: Candidate pathways and genes for prostate cancer: a meta-analysis of gene expression data
Source: BMC Med Genomics. 2009 Aug 4;2:48. doi: 10.1186/1755-8794-2-48 (PMC2731785; doi:10.1186/1755-8794-2-48)
Supplement: Additional file 2 — Description of how many genes to use for an assessment of functional clustering. Text representing discussion of many genes to use for an assessment of functional clustering. [file 1755-8794-2-48-S2.doc]

# Additional File 2.

# How many genes to use for an assessment of functional clustering

It is not clear how many genes one needs to include in functional annotation analysis. It is usually an arbitrary decision based on the researcher’s guess as to how many genes have importance. In this study, we tried to approach the problem more formally. We assumed that there should be an optimal number of genes to include into analysis. Indeed, if we included into analysis just the genes with the largest difference in gene expression level, the number of genes in the analysis would not be enough to make a statistically significant association between the top genes and specific pathways or molecular functions, even though all these genes are very likely to be functional and important for tumor progression. On the other hand, if we went beyond the top genes, there was a possibility that some of the genes would not be related to the phenotype of interest, in our case tumor progression. Including nonrelated genes in the analysis would dilute the signal and therefore decrease the statistical power to detect an association between the genes, pathways and molecular functions. Therefore, we expected to have an inverse U-type relationship between the numbers of associated pathways or functions and the number of genes in our analysis. The Supplementary Fig. 1 shows the relationship between the number of significant functions defined by Ingenuity and the number of genes in the analysis. We found that the number of functions significantly associated with the top genes was associated with the number of top genes such that it first increased and then decreased. When the number of significant functions increased, it occurred by adding functions, not replacing them. Based on the observed relation between the number of significant functions/pathways and the number of top genes, we decided to use the top 500 genes for functional annotation.

**Supplementary Figure 1.** Dependence between the number of functions and canonical pathways defined based on the top genes.
