# Supplementary material for: Candidate pathways and genes for prostate cancer: a meta-analysis of gene expression data
Source: BMC Med Genomics. 2009 Aug 4;2:48. doi: 10.1186/1755-8794-2-48 (PMC2731785; doi:10.1186/1755-8794-2-48)
Supplement: Additional file 3 — List of the top 500 genes differentially expressed between normal prostate and localized prostate tumor – NP-nMPC transition. The data provided represent the list of the top 500 genes differentially expressed between normal prostate and localized prostate tumor. [file 1755-8794-2-48-S3.doc]

Additional File 3.

**The top 500 genes differentially expressed between normal prostate and localized prostate tumor - NP-nMPC transition.**

| Gene_Symbol | Z-score | Direction | P-value |
| --- | --- | --- | --- |
| AMACR | 21.87 | Up | 3E-80 |
| MYO6 | 17.76 | Up | 5.55E-61 |
| UAP1 | 16.77 | Up | 2.47E-56 |
| FER1L3 | -15.91 | Down | 2.74E-52 |
| PTRF | -15.45 | Down | 3.57E-50 |
| CAV2 | -15.33 | Down | 1.36E-49 |
| PBX1 | -15.14 | Down | 1.1E-48 |
| SLC25A6 | 14.99 | Up | 5.19E-48 |
| SYNPO2 | -14.59 | Down | 3.84E-46 |
| CAMKK2 | 14.57 | Up | 4.75E-46 |
| RND3 | -14.39 | Down | 3.53E-45 |
| KIAA1450 | 14.29 | Up | 1.08E-44 |
| CALM1 | -14.00 | Down | 2.39E-43 |
| SOX4 | 13.98 | Up | 2.91E-43 |
| SEL1L | 13.96 | Up | 3.65E-43 |
| FGFR2 | -13.92 | Down | 5.32E-43 |
| RBPMS | -13.84 | Down | 1.36E-42 |
| C20orf199 | 13.74 | Up | 3.85E-42 |
| GSTM2 | -13.59 | Down | 1.87E-41 |
| DST | -13.48 | Down | 6.42E-41 |
| HPN | 13.41 | Up | 1.33E-40 |
| MYC | 13.38 | Up | 1.81E-40 |
| PAICS | 13.23 | Up | 9.83E-40 |
| SNAI2 | -13.11 | Down | 3.53E-39 |
| F5 | 12.94 | Up | 2.25E-38 |
| VCL | -12.93 | Down | 2.32E-38 |
| CALD1 | -12.90 | Down | 3.34E-38 |
| OGT | 12.82 | Up | 8.1E-38 |
| JOSD3 | 12.82 | Up | 8.26E-38 |
| MEIS2 | -12.81 | Down | 8.84E-38 |
| SGCB | -12.80 | Down | 1.02E-37 |
| MYH11 | -12.78 | Down | 1.26E-37 |
| ERG | 12.74 | Up | 1.79E-37 |
| DNAJC10 | 12.74 | Up | 1.81E-37 |
| EFS | -12.70 | Down | 2.83E-37 |
| ANXA2 | -12.67 | Down | 4.14E-37 |
| ATP2B4 | -12.66 | Down | 4.4E-37 |
| FHL1 | -12.55 | Down | 1.48E-36 |
| MCCC2 | 12.54 | Up | 1.62E-36 |
| TIMP3 | -12.52 | Down | 2E-36 |
| GSTP1 | -12.51 | Down | 2.15E-36 |
| F2R | 12.45 | Up | 4.14E-36 |
| TACSTD1 | 12.44 | Up | 4.86E-36 |
| RGS10 | 12.43 | Up | 5.35E-36 |
| GJB1 | 12.42 | Up | 6.1E-36 |
| CTSB | -12.41 | Down | 6.54E-36 |
| ABCC4 | 12.38 | Up | 8.8E-36 |
| SCRN1 | -12.36 | Down | 1.18E-35 |
| COL4A6 | -12.31 | Down | 2E-35 |
| TGFBR3 | -12.21 | Down | 6E-35 |
| CDC42EP4 | -12.17 | Down | 9.23E-35 |
| TACC1 | -12.12 | Down | 1.54E-34 |
| LGALS3BP | -12.09 | Down | 2.05E-34 |
| GAS1 | -12.06 | Down | 3.04E-34 |
| DDR2 | -12.06 | Down | 3.05E-34 |
| CAV1 | -11.96 | Down | 8.17E-34 |
| TPM1 | -11.95 | Down | 9.74E-34 |
| BICD1 | 11.93 | Up | 1.14E-33 |
| SLC14A1 | -11.82 | Down | 3.78E-33 |
| GPRC5B | -11.74 | Down | 9.15E-33 |
| ZNF532 | -11.73 | Down | 9.77E-33 |
| SH3RF1 | 11.71 | Up | 1.32E-32 |
| GLYATL1 | 11.68 | Up | 1.79E-32 |
| UCK2 | 11.63 | Up | 2.94E-32 |
| DAPK1 | 11.59 | Up | 4.68E-32 |
| RPL22L1 | 11.57 | Up | 5.8E-32 |
| DKFZP564O0823 | -11.56 | Down | 6.23E-32 |
| PDLIM5 | 11.56 | Up | 6.29E-32 |
| FCGRT | -11.54 | Down | 7.6E-32 |
| ZBTB4 | -11.52 | Down | 9.99E-32 |
| ENAH | -11.41 | Down | 3.29E-31 |
| NBL1 | -11.40 | Down | 3.61E-31 |
| GATM | -11.38 | Down | 4.27E-31 |
| GJA1 | -11.38 | Down | 4.38E-31 |
| GOLM1 | 11.38 | Up | 4.66E-31 |
| HMGN4 | -11.36 | Down | 5.26E-31 |
| POLR2H | 11.34 | Up | 6.87E-31 |
| DNAH5 | 11.32 | Up | 8.78E-31 |
| ATP8A1 | 11.30 | Up | 1.09E-30 |
| SEC16A | 11.29 | Up | 1.22E-30 |
| HSPB8 | -11.24 | Down | 2.13E-30 |
| NME1 | 11.23 | Up | 2.14E-30 |
| PLP2 | -11.17 | Down | 4.27E-30 |
| E2F5 | 11.17 | Up | 4.42E-30 |
| ACTN1 | -11.13 | Down | 6.88E-30 |
| TRIM27 | 11.12 | Up | 7.44E-30 |
| MBNL2 | -11.11 | Down | 8.66E-30 |
| FADS1 | -11.10 | Down | 8.72E-30 |
| SMTN | -11.09 | Down | 9.76E-30 |
| SIM2 | 11.02 | Up | 2.21E-29 |
| GUCY1A3 | 11.00 | Up | 2.76E-29 |
| TARP | 10.99 | Up | 3.13E-29 |
| PRSS23 | -10.98 | Down | 3.49E-29 |
| CADM1 | 10.92 | Up | 6.5E-29 |
| SEC23A | -10.89 | Down | 8.92E-29 |
| MEIS1 | -10.89 | Down | 9.29E-29 |
| ST5 | -10.87 | Down | 1.07E-28 |
| RCAN2 | -10.86 | Down | 1.27E-28 |
| ZNF217 | 10.82 | Up | 1.83E-28 |
| CRYAB | -10.82 | Down | 1.95E-28 |
| DKK3 | -10.81 | Down | 2.09E-28 |
| OAT | -10.77 | Down | 3.19E-28 |
| UGDH | 10.76 | Up | 3.46E-28 |
| SLC43A1 | 10.76 | Up | 3.61E-28 |
| CDC42EP3 | -10.72 | Down | 5.55E-28 |
| ANG | -10.69 | Down | 7.58E-28 |
| SPG20 | -10.68 | Down | 8.78E-28 |
| ACACA | 10.64 | Up | 1.25E-27 |
| PTP4A2 | -10.64 | Down | 1.37E-27 |
| ID4 | -10.62 | Down | 1.58E-27 |
| SAMD5 | 10.60 | Up | 2.04E-27 |
| AMOT | -10.59 | Down | 2.32E-27 |
| GCNT1 | 10.57 | Up | 2.9E-27 |
| GPR160 | 10.55 | Up | 3.29E-27 |
| SYNJ2BP | 10.55 | Up | 3.42E-27 |
| MYLK | -10.55 | Down | 3.47E-27 |
| GNB2L1 | 10.55 | Up | 3.52E-27 |
| TAGLN | -10.55 | Down | 3.63E-27 |
| CKS2 | 10.53 | Up | 4.13E-27 |
| CYP3A5 | -10.52 | Down | 4.7E-27 |
| GSTM4 | -10.52 | Down | 4.94E-27 |
| DUSP3 | -10.48 | Down | 7.21E-27 |
| PMP22 | -10.46 | Down | 8.83E-27 |
| MPZL2 | -10.45 | Down | 1.04E-26 |
| C6orf108 | 10.43 | Up | 1.26E-26 |
| C7orf24 | 10.43 | Up | 1.28E-26 |
| METTL7A | -10.42 | Down | 1.34E-26 |
| TPM2 | -10.42 | Down | 1.48E-26 |
| ITM2C | -10.39 | Down | 1.93E-26 |
| REPS2 | 10.39 | Up | 1.96E-26 |
| TRIP6 | -10.38 | Down | 2.23E-26 |
| GATA3 | -10.36 | Down | 2.63E-26 |
| GABRB3 | 10.34 | Up | 3.25E-26 |
| ITGA2 | -10.33 | Down | 3.65E-26 |
| ABHD2 | 10.33 | Up | 3.9E-26 |
| MAL2 | 10.30 | Up | 4.92E-26 |
| GSTM5 | -10.27 | Down | 7.41E-26 |
| LOC152485 | -10.26 | Down | 7.65E-26 |
| UTRN | 10.25 | Up | 8.93E-26 |
| ANK3 | 10.24 | Up | 1E-25 |
| C9orf91 | 10.24 | Up | 1.01E-25 |
| THBS4 | 10.20 | Up | 1.55E-25 |
| NCI_CGAP_Pr2 | 10.20 | Up | 1.58E-25 |
| SGEF | 10.20 | Up | 1.58E-25 |
| LDB3 | -10.19 | Down | 1.66E-25 |
| ST6GALNAC2 | -10.18 | Down | 1.81E-25 |
| IGF1 | -10.16 | Down | 2.27E-25 |
| PCGF3 | 10.15 | Up | 2.47E-25 |
| PPAP2B | -10.13 | Down | 3.36E-25 |
| CLU | -10.09 | Down | 5.04E-25 |
| GALNT3 | 10.07 | Up | 6.41E-25 |
| TRIM29 | -10.06 | Down | 7.2E-25 |
| RCC1 | 10.05 | Up | 7.71E-25 |
| CMTM4 | 10.05 | Up | 7.83E-25 |
| DKC1 | 10.05 | Up | 7.91E-25 |
| SERP1 | 10.04 | Up | 8.51E-25 |
| IL6ST | -10.01 | Down | 1.21E-24 |
| CNN1 | -9.99 | Down | 1.47E-24 |
| GAS6 | -9.98 | Down | 1.57E-24 |
| KCNMB1 | -9.97 | Down | 1.81E-24 |
| NT5E | -9.96 | Down | 1.99E-24 |
| ATP7B | 9.95 | Up | 2.3E-24 |
| SMAD3 | -9.94 | Down | 2.42E-24 |
| SMARCD3 | -9.93 | Down | 2.87E-24 |
| ANTXR1 | -9.92 | Down | 2.96E-24 |
| PLA1A | 9.92 | Up | 3.04E-24 |
| ACTG2 | -9.89 | Down | 4.12E-24 |
| PRDX4 | 9.86 | Up | 5.99E-24 |
| FGFR1 | -9.85 | Down | 6.55E-24 |
| ST14 | 9.85 | Up | 6.94E-24 |
| IMPDH2 | 9.83 | Up | 8.25E-24 |
| MME | -9.83 | Down | 8.42E-24 |
| MRPL42 | 9.83 | Up | 8.6E-24 |
| MANEAL | 9.83 | Up | 8.6E-24 |
| MAP7 | 9.82 | Up | 9.06E-24 |
| LMOD1 | -9.79 | Down | 1.29E-23 |
| GSTM3 | -9.78 | Down | 1.35E-23 |
| MAST4 | -9.78 | Down | 1.35E-23 |
| ICA1 | 9.78 | Up | 1.41E-23 |
| TP63 | -9.76 | Down | 1.74E-23 |
| RICS | 9.75 | Up | 1.92E-23 |
| GPM6B | -9.74 | Down | 2.06E-23 |
| SMARCC1 | 9.74 | Up | 2.2E-23 |
| ALCAM | 9.73 | Up | 2.33E-23 |
| AKAP12 | -9.73 | Down | 2.4E-23 |
| LYCAT | 9.72 | Up | 2.78E-23 |
| TMEM47 | -9.69 | Down | 3.61E-23 |
| septin 11 | 9.68 | Up | 3.96E-23 |
| FOLH1 | 9.67 | Up | 4.53E-23 |
| ACSS3 | -9.64 | Down | 6.35E-23 |
| ZNF655 | -9.64 | Down | 6.44E-23 |
| C20orf74 | 9.64 | Up | 6.51E-23 |
| FHL2 | -9.64 | Down | 6.72E-23 |
| PPIB | 9.62 | Up | 7.9E-23 |
| INSM1 | 9.61 | Up | 8.66E-23 |
| COX7A1 | -9.61 | Down | 8.84E-23 |
| HIPK2 | 9.59 | Up | 1.07E-22 |
| EEF2 | 9.59 | Up | 1.15E-22 |
| ATP6V1G1 | 9.59 | Up | 1.16E-22 |
| SBK1 | 9.58 | Up | 1.16E-22 |
| TMSL8 | 9.58 | Up | 1.22E-22 |
| ENC1 | 9.57 | Up | 1.36E-22 |
| PPP1R12B | -9.56 | Down | 1.52E-22 |
| MXRA7 | -9.56 | Down | 1.56E-22 |
| CLIC4 | -9.55 | Down | 1.63E-22 |
| RAB34 | -9.53 | Down | 2.09E-22 |
| COL9A2 | 9.52 | Up | 2.26E-22 |
| C9orf3 | -9.51 | Down | 2.58E-22 |
| DPYSL3 | -9.47 | Down | 3.8E-22 |
| ZCCHC6 | 9.47 | Up | 3.88E-22 |
| RAB11A | 9.46 | Up | 4.54E-22 |
| C10orf116 | -9.45 | Down | 5.07E-22 |
| AOC3 | -9.43 | Down | 6.14E-22 |
| MARCKSL1 | 9.41 | Up | 7.58E-22 |
| TSPAN13 | 9.41 | Up | 7.61E-22 |
| NCI_CGAP_Pr1 | 9.37 | Up | 1.14E-21 |
| DMKN | -9.36 | Down | 1.3E-21 |
| LPIN1 | -9.34 | Down | 1.55E-21 |
| MON1B | 9.34 | Up | 1.62E-21 |
| BDH2 | -9.33 | Down | 1.8E-21 |
| VPS37A | -9.33 | Down | 1.87E-21 |
| ATP11B | 9.33 | Up | 1.9E-21 |
| KLHL8 | 9.33 | Up | 1.9E-21 |
| PDIA5 | 9.31 | Up | 2.17E-21 |
| PCP4 | -9.30 | Down | 2.45E-21 |
| EFEMP2 | -9.29 | Down | 2.85E-21 |
| ATP11A | 9.28 | Up | 2.95E-21 |
| CAMK2G | -9.28 | Down | 3.14E-21 |
| SND1 | 9.27 | Up | 3.29E-21 |
| KIAA1727 | -9.27 | Down | 3.36E-21 |
| GBP2 | -9.26 | Down | 3.88E-21 |
| EIF3B | 9.26 | Up | 4.07E-21 |
| PTN | -9.21 | Down | 6.53E-21 |
| MAP9 | 9.20 | Up | 7.11E-21 |
| AURKA | 9.20 | Up | 7.59E-21 |
| TSTA3 | 9.19 | Up | 7.83E-21 |
| CBX3 | 9.19 | Up | 8.31E-21 |
| ACTA2 | -9.18 | Down | 9.41E-21 |
| MAP1B | -9.18 | Down | 9.53E-21 |
| ASPN | 9.18 | Up | 9.59E-21 |
| BPHL | 9.17 | Up | 1.04E-20 |
| EPAS1 | -9.16 | Down | 1.19E-20 |
| GALNT1 | 9.14 | Up | 1.43E-20 |
| YAP1 | -9.14 | Down | 1.43E-20 |
| ANGPT1 | -9.13 | Down | 1.54E-20 |
| MYLIP | 9.13 | Up | 1.64E-20 |
| PLEKHC1 | -9.13 | Down | 1.65E-20 |
| TncRNA | 9.13 | Up | 1.65E-20 |
| FAM84B | 9.12 | Up | 1.68E-20 |
| C12orf51 | 9.12 | Up | 1.69E-20 |
| RCC2 | 9.11 | Up | 1.98E-20 |
| SH3BGRL2 | -9.10 | Down | 2.11E-20 |
| ANAPC5 | 9.10 | Up | 2.26E-20 |
| PPP1R12A | -9.09 | Down | 2.45E-20 |
| PMS2L1 | 9.09 | Up | 2.53E-20 |
| SLC35F5 | 9.07 | Up | 2.88E-20 |
| SRP72 | 9.07 | Up | 3.06E-20 |
| MAT2B | -9.06 | Down | 3.51E-20 |
| EIF4A1 | 9.05 | Up | 3.56E-20 |
| GABARAPL1 | -9.05 | Down | 3.9E-20 |
| ZDHHC9 | 9.04 | Up | 3.99E-20 |
| RBMS1 | -9.04 | Down | 4.15E-20 |
| ZNF516 | -9.01 | Down | 5.46E-20 |
| SRPX | -9.01 | Down | 5.68E-20 |
| GPD1L | -9.00 | Down | 6.24E-20 |
| AQP3 | -8.99 | Down | 7E-20 |
| PDIA3 | 8.99 | Up | 7.15E-20 |
| RPL29 | 8.97 | Up | 8.64E-20 |
| IFITM1 | -8.97 | Down | 8.92E-20 |
| FLNC | -8.96 | Down | 1E-19 |
| RGL2 | 8.94 | Up | 1.18E-19 |
| MAF | -8.94 | Down | 1.19E-19 |
| P4HB | 8.93 | Up | 1.31E-19 |
| NME2 | 8.93 | Up | 1.39E-19 |
| B4GALT3 | 8.92 | Up | 1.46E-19 |
| SERPINB1 | -8.92 | Down | 1.59E-19 |
| CD59 | -8.91 | Down | 1.76E-19 |
| ITPR3 | 8.91 | Up | 1.78E-19 |
| OCLN | 8.90 | Up | 1.92E-19 |
| PALLD | -8.89 | Down | 2E-19 |
| SLMAP | -8.89 | Down | 2.01E-19 |
| COL17A1 | -8.89 | Down | 2.05E-19 |
| CSRP1 | -8.88 | Down | 2.36E-19 |
| SDC2 | -8.87 | Down | 2.66E-19 |
| FABP5 | 8.86 | Up | 2.92E-19 |
| F2RL1 | 8.86 | Up | 3.04E-19 |
| GNG11 | -8.85 | Down | 3.24E-19 |
| ERBB3 | 8.84 | Up | 3.5E-19 |
| LOC257407 | 8.83 | Up | 4.14E-19 |
| STK36 | 8.83 | Up | 4.14E-19 |
| RBM9 | -8.82 | Down | 4.42E-19 |
| CNTN1 | -8.82 | Down | 4.64E-19 |
| IFITM2 | -8.81 | Down | 4.72E-19 |
| TMED3 | 8.81 | Up | 4.86E-19 |
| BTG3 | -8.81 | Down | 5.07E-19 |
| RPL14 | 8.80 | Up | 5.72E-19 |
| SDK1 | 8.79 | Up | 5.87E-19 |
| RPLP0 | 8.79 | Up | 6.01E-19 |
| MYL9 | -8.78 | Down | 6.79E-19 |
| PARVA | -8.78 | Down | 7.13E-19 |
| GSN | -8.78 | Down | 7.15E-19 |
| MAPK6 | 8.78 | Up | 7.18E-19 |
| MARVELD2 | 8.77 | Up | 7.56E-19 |
| NEDD4L | 8.77 | Up | 7.86E-19 |
| SPON1 | -8.76 | Down | 8.2E-19 |
| CDKN1C | -8.76 | Down | 8.99E-19 |
| PIK3R1 | -8.75 | Down | 9.47E-19 |
| TOM1L1 | 8.73 | Up | 1.19E-18 |
| TUBA4A | -8.73 | Down | 1.2E-18 |
| ETV5 | -8.73 | Down | 1.2E-18 |
| CNPY2 | 8.72 | Up | 1.29E-18 |
| IRS1 | -8.71 | Down | 1.44E-18 |
| GUSB | 8.70 | Up | 1.56E-18 |
| AOX1 | -8.70 | Down | 1.58E-18 |
| KCNAB1 | -8.70 | Down | 1.71E-18 |
| ACSF2 | -8.69 | Down | 1.78E-18 |
| ALDH1A2 | -8.69 | Down | 1.91E-18 |
| FAM107A | -8.68 | Down | 1.97E-18 |
| TLE2 | -8.68 | Down | 2E-18 |
| ARMET | 8.66 | Up | 2.43E-18 |
| TRAM2 | 8.65 | Up | 2.7E-18 |
| CAPZB | -8.65 | Down | 2.77E-18 |
| ANTXR2 | -8.64 | Down | 3.28E-18 |
| HMG20B | 8.63 | Up | 3.55E-18 |
| CBR4 | 8.62 | Up | 3.82E-18 |
| EDG2 | -8.62 | Down | 3.91E-18 |
| FZD7 | -8.61 | Down | 4.23E-18 |
| TOR1AIP1 | -8.60 | Down | 4.57E-18 |
| ISCU | -8.60 | Down | 4.63E-18 |
| PLA2G7 | 8.60 | Up | 4.77E-18 |
| PRKCB1 | -8.60 | Down | 4.89E-18 |
| MEIS3P1 | -8.59 | Down | 5.19E-18 |
| KCNH2 | -8.58 | Down | 5.91E-18 |
| RTN4 | -8.58 | Down | 6.01E-18 |
| HEXIM1 | -8.57 | Down | 6.49E-18 |
| NNT | -8.57 | Down | 6.89E-18 |
| C4orf14 | 8.56 | Up | 7.4E-18 |
| BGN | 8.56 | Up | 7.69E-18 |
| FZD8 | 8.56 | Up | 7.72E-18 |
| TGFB1I1 | -8.55 | Down | 8.01E-18 |
| ETS2 | -8.54 | Down | 9.59E-18 |
| PGRMC1 | -8.52 | Down | 1.13E-17 |
| RAB31 | -8.52 | Down | 1.14E-17 |
| BIN1 | -8.52 | Down | 1.16E-17 |
| COL6A1 | -8.50 | Down | 1.4E-17 |
| CLDN8 | 8.49 | Up | 1.58E-17 |
| PNPT1 | 8.48 | Up | 1.69E-17 |
| SPARCL1 | -8.48 | Down | 1.79E-17 |
| MIPEP | 8.47 | Up | 2.02E-17 |
| MT1X | -8.45 | Down | 2.39E-17 |
| CASP1 | -8.45 | Down | 2.4E-17 |
| WFDC2 | -8.45 | Down | 2.53E-17 |
| RAP1A | -8.44 | Down | 2.72E-17 |
| MTHFD2 | 8.43 | Up | 2.97E-17 |
| RBM35A | 8.43 | Up | 2.98E-17 |
| JAM3 | -8.43 | Down | 3.15E-17 |
| RAB17 | 8.42 | Up | 3.35E-17 |
| PPP2CB | -8.41 | Down | 3.58E-17 |
| WWTR1 | -8.41 | Down | 3.76E-17 |
| CREM | -8.41 | Down | 3.83E-17 |
| GNPNAT1 | 8.40 | Up | 4.07E-17 |
| TRAP1 | 8.39 | Up | 4.69E-17 |
| APRT | 8.39 | Up | 4.84E-17 |
| ECHDC1 | -8.38 | Down | 4.97E-17 |
| ADH5 | -8.38 | Down | 4.98E-17 |
| SGCE | -8.38 | Down | 5.22E-17 |
| P2RX4 | 8.38 | Up | 5.3E-17 |
| GCS1 | 8.38 | Up | 5.36E-17 |
| ANKRD15 | -8.37 | Down | 5.48E-17 |
| C2orf43 | -8.37 | Down | 5.58E-17 |
| PTPRN2 | 8.37 | Up | 5.86E-17 |
| PRKCA | -8.36 | Down | 6.15E-17 |
| SLC45A2 | 8.36 | Up | 6.22E-17 |
| LOC391356 | 8.34 | Up | 7.74E-17 |
| PPP1R3C | -8.34 | Down | 8.08E-17 |
| STRA13 | 8.34 | Up | 8.14E-17 |
| NR3C1 | -8.32 | Down | 9.63E-17 |
| KIAA1128 | -8.32 | Down | 1.01E-16 |
| DNAJB5 | -8.31 | Down | 1.05E-16 |
| TGFB2 | -8.30 | Down | 1.17E-16 |
| SYNGR1 | -8.30 | Down | 1.2E-16 |
| DDOST | 8.30 | Up | 1.21E-16 |
| CDC14B | -8.30 | Down | 1.28E-16 |
| MSRB3 | -8.29 | Down | 1.35E-16 |
| TGFB3 | -8.29 | Down | 1.44E-16 |
| NET1 | 8.27 | Up | 1.6E-16 |
| ANXA1 | -8.27 | Down | 1.61E-16 |
| OPTN | -8.27 | Down | 1.62E-16 |
| CAMK2D | -8.27 | Down | 1.65E-16 |
| FLRT3 | -8.27 | Down | 1.66E-16 |
| EDNRA | -8.27 | Down | 1.76E-16 |
| ZMYND8 | 8.26 | Up | 1.91E-16 |
| SLC2A5 | -8.25 | Down | 2E-16 |
| CPSF6 | 8.25 | Up | 2.17E-16 |
| ANKH | -8.24 | Down | 2.28E-16 |
| RPS16 | 8.23 | Up | 2.53E-16 |
| HLTF | 8.23 | Up | 2.63E-16 |
| hCG_40738 | 8.22 | Up | 2.77E-16 |
| TOX3 | 8.21 | Up | 3.06E-16 |
| TBL1X | -8.21 | Down | 3.15E-16 |
| NUP210 | 8.20 | Up | 3.6E-16 |
| THOC2 | 8.19 | Up | 4.19E-16 |
| JAK1 | -8.18 | Down | 4.54E-16 |
| KRT18 | 8.17 | Up | 4.8E-16 |
| RPS7 | 8.17 | Up | 4.81E-16 |
| ST3GAL5 | -8.16 | Down | 5.35E-16 |
| NCI_CGAP_Pr3 | 8.15 | Up | 6.06E-16 |
| ITGA3 | -8.15 | Down | 6.15E-16 |
| FLNA | -8.14 | Down | 6.58E-16 |
| CSRP2 | -8.14 | Down | 6.97E-16 |
| LASS6 | 8.14 | Up | 7.07E-16 |
| C4orf18 | 8.13 | Up | 7.44E-16 |
| CANX | 8.13 | Up | 7.59E-16 |
| ZNF350 | 8.12 | Up | 8.71E-16 |
| COPZ2 | -8.12 | Down | 8.95E-16 |
| LOC389831 | -8.10 | Down | 1.06E-15 |
| CRTAP | -8.10 | Down | 1.08E-15 |
| ITSN1 | -8.10 | Down | 1.08E-15 |
| IQGAP2 | 8.10 | Up | 1.09E-15 |
| BRP44 | 8.09 | Up | 1.2E-15 |
| LYPLA1 | 8.08 | Up | 1.28E-15 |
| HDAC1 | 8.08 | Up | 1.32E-15 |
| IFITM3 | -8.07 | Down | 1.5E-15 |
| SCHIP1 | -8.06 | Down | 1.65E-15 |
| ID1 | -8.05 | Down | 1.77E-15 |
| CYP4B1 | -8.04 | Down | 2.12E-15 |
| RUVBL1 | 8.02 | Up | 2.63E-15 |
| ALG8 | 8.00 | Up | 3.01E-15 |
| SSR2 | 8.00 | Up | 3.22E-15 |
| TSPAN1 | 7.99 | Up | 3.52E-15 |
| LPP | -7.99 | Down | 3.65E-15 |
| PLAGL1 | -7.98 | Down | 3.71E-15 |
| KIAA0999 | -7.98 | Down | 3.86E-15 |
| SRM | 7.98 | Up | 3.88E-15 |
| IGF2 | -7.98 | Down | 3.95E-15 |
| CRISPLD2 | -7.95 | Down | 5.17E-15 |
| ITPR2 | 7.95 | Up | 5.51E-15 |
| C16orf5 | -7.95 | Down | 5.56E-15 |
| SLC35A3 | 7.94 | Up | 5.76E-15 |
| ADRB2 | 7.94 | Up | 6E-15 |
| HIST1H4H | 7.94 | Up | 6.19E-15 |
| RPL31 | 7.93 | Up | 6.46E-15 |
| OAS2 | 7.93 | Up | 6.56E-15 |
| PYGL | -7.93 | Down | 6.67E-15 |
| UBE2E3 | 7.93 | Up | 6.87E-15 |
| MAPRE1 | -7.92 | Down | 6.99E-15 |
| HSD17B4 | 7.90 | Up | 8.81E-15 |
| C1orf59 | 7.90 | Up | 9.48E-15 |
| LHFPL2 | -7.89 | Down | 9.92E-15 |
| APOC1 | 7.89 | Up | 1.03E-14 |
| HNRPH3 | -7.89 | Down | 1.05E-14 |
| STAT3 | -7.87 | Down | 1.23E-14 |
| CLIP4 | -7.87 | Down | 1.26E-14 |
| FMO5 | 7.87 | Up | 1.29E-14 |
| MPP6 | 7.86 | Up | 1.41E-14 |
| EMP3 | -7.86 | Down | 1.45E-14 |
| XPO6 | 7.84 | Up | 1.73E-14 |
| PUS7 | 7.83 | Up | 1.91E-14 |
| RPS19 | 7.83 | Up | 2.02E-14 |
| TRPS1 | -7.83 | Down | 2.04E-14 |
| CYFIP2 | 7.82 | Up | 2.28E-14 |
| NLK | 7.81 | Up | 2.32E-14 |
| INSIG1 | -7.81 | Down | 2.52E-14 |
| EGR2 | 7.81 | Up | 2.55E-14 |
| ARID1A | 7.80 | Up | 2.63E-14 |
| ARHGAP24 | -7.80 | Down | 2.71E-14 |
| LOC202451 | 7.80 | Up | 2.79E-14 |
| PDE4D | -7.79 | Down | 2.87E-14 |
| SNRPD2 | 7.79 | Up | 3.04E-14 |
| STIL | 7.79 | Up | 3.1E-14 |
| SVIL | -7.79 | Down | 3.12E-14 |
| MBOAT2 | 7.79 | Up | 3.14E-14 |
| NA | -7.78 | Down | 3.34E-14 |
| PPP3CA | 7.78 | Up | 3.49E-14 |
| KPNA3 | -7.77 | Down | 3.53E-14 |
| RPESP | -7.77 | Down | 3.83E-14 |
| PPM2C | 7.77 | Up | 3.93E-14 |
| TCF7L1 | -7.76 | Down | 4.06E-14 |
| CAP2 | -7.76 | Down | 4.14E-14 |
| JARID1B | 7.75 | Up | 4.41E-14 |
| GPX2 | -7.75 | Down | 4.6E-14 |
| NCAM1 | -7.75 | Down | 4.71E-14 |
| NACA | 7.75 | Up | 4.78E-14 |
| EPB41L3 | -7.75 | Down | 4.83E-14 |
| PGCP | -7.75 | Down | 4.85E-14 |
| MET | -7.74 | Down | 5.04E-14 |
| TEAD3 | -7.73 | Down | 5.47E-14 |
| RAB3IP | 7.73 | Up | 5.64E-14 |
| DPT | -7.73 | Down | 5.77E-14 |
| ZNRF2 | 7.72 | Up | 6.23E-14 |
| ZMPSTE24 | 7.72 | Up | 6.56E-14 |
| FBXL7 | -7.72 | Down | 6.63E-14 |
| RCL1 | 7.71 | Up | 6.88E-14 |
| WDR68 | 7.71 | Up | 6.91E-14 |
| MYL6 | -7.71 | Down | 7.17E-14 |
| NID1 | -7.69 | Down | 8.44E-14 |
| LAPTM4B | -7.69 | Down | 8.73E-14 |
| PKIB | 7.69 | Up | 8.93E-14 |
| MAOB | -7.69 | Down | 9.04E-14 |
| FAM83H | 7.69 | Up | 9.19E-14 |
