# Supplementary figures and images for: Candidate pathways and genes for prostate cancer: a meta-analysis of gene expression data
Source: BMC Med Genomics. 2009 Aug 4;2:48. doi: 10.1186/1755-8794-2-48 (PMC2731785; doi:10.1186/1755-8794-2-48)

## FOCAL ADHESION

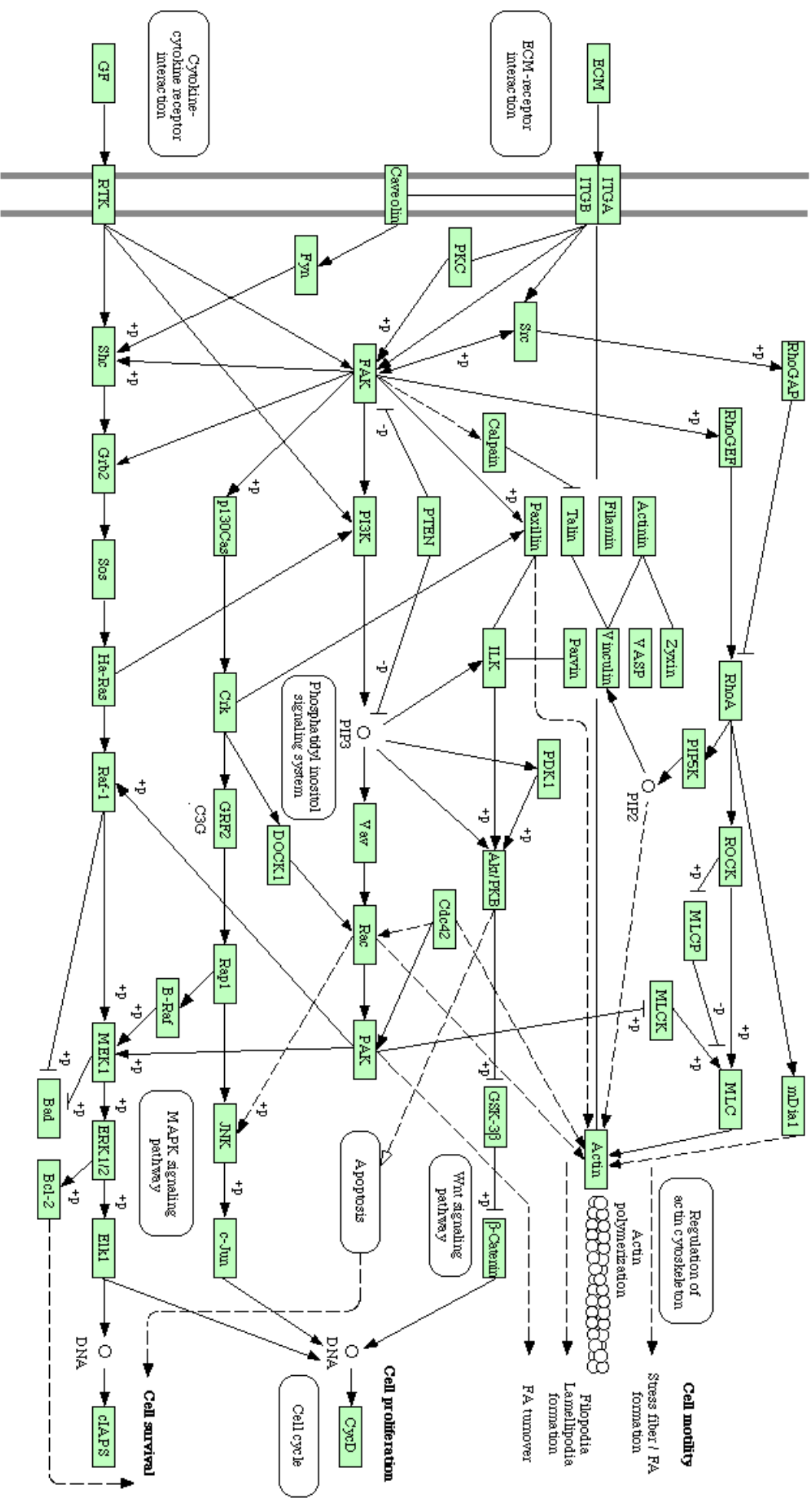

Supplement: Additional file 4 — Schematics of cell adhesion pathway (according to the Kyoto Encyclopedia of Genes and Genomes). Picture representing cell adhesion pathway. [file 1755-8794-2-48-S4.pdf]
