# Supplementary material for: Candidate pathways and genes for prostate cancer: a meta-analysis of gene expression data
Source: BMC Med Genomics. 2009 Aug 4;2:48. doi: 10.1186/1755-8794-2-48 (PMC2731785; doi:10.1186/1755-8794-2-48)
Supplement: Additional file 5 — List of genes for Table 2 "Canonical pathways significant in the transition from normal prostate to localized prostate cancer (functional annotation by Ingenuity software)". The data provided represent the list of genes for Table 2. [file 1755-8794-2-48-S5.doc]

Additional File 8.

**List of genes for the Table 2 “Canonical pathways significant in the transition from normal prostate to localized prostate cancer (functional annotation by Ingenuity software)”.**

| **Pathway** | **Genes** |
| --- | --- |
| Calcium Signaling | TPM1, TP63, MYL6, ITPR2, RCAN2, HDAC1 (includes EG:3065), ACTA2 (includes EG:59), TPM2, MYH11, RAP1A, MYL9 (includes EG:10398), CAMK2D, ITPR3, CAMKK2, ATP2B4, PPP3CA, CALM1, CAMK2G |
| NRF2-mediated Oxidative Stress Response | GSTP1, GSTM5, PPIB, PIK3R1, GSTM3 (includes EG:2947), ACTA2 (includes EG:59), MAF, HSPB8, DNAJC10, GSTM2, GPX2, GSTM4, ACTG2, AOX1, DNAJB5, PRKCB1, PRKCA |
| Actin Cytoskeleton Signaling | F2R, MYL6, PIK3R1, ITGA2, ACTA2 (includes EG:59), MYH11, GSN, ITGA3, MYLK, MYL9 (includes EG:10398), IQGAP2, CYFIP2, PPP1R12B, PPP1R12A, VCL, ACTG2, ACTN1 |
| Tight Junction Signaling | MYL6, ACTA2 (includes EG:59), MYH11, OCLN, MYL9 (includes EG:10398), MYLK, CPSF6, PPP2CB, CLDN8, JAM3, TGFB3, TGFB2, VCL, ACTG2 |
| Synaptic Long Term Potentiation | CAMK2D, ITPR2, PPP1R3C, ITPR3, PPP1R12A, RAP1A, PPP3CA, CALM1, CAMK2G, PRKCA, PRKCB1 |
| Hepatic Fibrosis / Hepatic Stellate Cell Activation | MET, MYL9 (includes EG:10398), MYL6, IGF1, FGFR1, SMAD3, ACTA2 (includes EG:59), TGFB3, TGFB2, FGFR2, EDNRA, MYH11 |
| Integrin Signaling | PARVA, PIK3R1, ITGA2, ACTA2 (includes EG:59), ITGA3, RAP1A, MYLK, RND3, TSPAN1, CAV1, PPP1R12B, PPP1R12A, VCL, ACTG2, ACTN1 |
| EGF Signaling | JAK1, ITPR2, PIK3R1, ITPR3, STAT3, PRKCA |
| GM-CSF Signaling | CAMK2D, PIK3R1, GNB2L1, STAT3, PPP3CA, CAMK2G, PRKCB1 |
| Regulation of Actin-based Motility by Rho | MYLK, MYL6, RND3, ACTA2 (includes EG:59), PPP1R12B, PPP1R12A, ACTG2, GSN |
| Wnt/beta-catenin Signaling | SOX4, MYC, FZD8, PPP2CB, GJA1, NLK, DKK3, HDAC1 (includes EG:3065), TGFB3, TGFB2, TCF7L1, FZD7 |
| PDGF Signaling | MYC, JAK1, PIK3R1, CAV1, STAT3, PRKCA, PRKCB1 |
| Xenobiotic Metabolism Signaling | GSTP1, GSTM5, PIK3R1, GSTM3 (includes EG:2947), MAF, FMO5, CYP3A5, PPP2CB, MAOB, GSTM2, CAMK2D, ALDH1A2, GSTM4, PRKCB1, CAMK2G, PRKCA |
| Chemokine Signaling | CAMK2D, PPP1R12B, PPP1R12A, CALM1, CAMK2G, PRKCA, PRKCB1 |
| Arginine and Proline Metabolism | AOC3, SRM, MAOB, P4HB, OAT, ALDH1A2, GATM |
| Cell Cycle: G1/S Checkpoint Regulation | MYC, SMAD3, HDAC1 (includes EG:3065), TGFB3, E2F5, TGFB2 |
| ERK/MAPK Signaling | MYC, PPP2CB, PPP1R3C, PIK3R1, ITGA2, ETS2, PPP1R12A, STAT3, ITGA3, RAP1A, PRKCA, PRKCB1 |
| cAMP-mediated Signaling | AKAP12, CREM, CAMK2D, PKIB, RGS10, STAT3, PDE4D, RAP1A, PPP3CA, CALM1, CAMK2G, ADRB2 |
| VEGF Signaling | PIK3R1, ACTA2 (includes EG:59), VCL, ACTG2, ACTN1, PRKCA, PRKCB1 |
| Leukocyte Extravasation Signaling | TIMP3, MYL6, CLDN8, JAM3, PIK3R1, ACTA2 (includes EG:59), VCL, ACTG2, RAP1A, ACTN1, PRKCA, PRKCB1 |
| Neuregulin Signaling | MYC, PIK3R1, ITGA2, ERBB3, ITGA3, PRKCA, PRKCB1 |
| Nitric Oxide Signaling in the Cardiovascular System | GUCY1A3, ITPR2, PIK3R1, ITPR3, CAV1, CALM1 |
| Glutathione Metabolism | GSTP1, GSTM2, GSTM5, GSTM3 (includes EG:2947), GPX2, GSTM4 |
| LPS/IL-1 Mediated Inhibition of RXR Function | APOC1, GSTP1, MAOB, GSTM2, FABP5, GSTM5, GSTM3 (includes EG:2947), ALDH1A2, GSTM4, FMO5, ABCC4, CYP3A5 |
| Purine Metabolism | NME1, GUCY1A3, NME2, PAICS, RUVBL1, PDE4D, APRT, ATP11B, MPP6, IMPDH2, NT5E, PNPT1, POLR2H, ATP6V1G1, AOX1, HLTF |
| Fatty Acid Biosynthesis | ACACA, MCCC2 |
| Aryl Hydrocarbon Receptor Signaling | MYC, GSTP1, GSTM2, GSTM5, GSTM3 (includes EG:2947), ALDH1A2, TGFB3, GSTM4, TGFB2 |
| Glucocorticoid Receptor Signaling | JAK1, PIK3R1, SMAD3, PBX1, STAT3, NR3C1, ANXA1, TGFB2, TGFB3, POLR2H, CDKN1C, HLTF, PPP3CA, ADRB2 |
