# Supplementary material for: Candidate pathways and genes for prostate cancer: a meta-analysis of gene expression data
Source: BMC Med Genomics. 2009 Aug 4;2:48. doi: 10.1186/1755-8794-2-48 (PMC2731785; doi:10.1186/1755-8794-2-48)
Supplement: Additional file 6 — List of the top 500 differentially expressed genes in the transition from localized to metastatic prostate cancer – nMPC-MPC transition. The data provided represent the list of the top 500 differentially expressed genes in the transition from localized to metastatic prostate cancer. [file 1755-8794-2-48-S6.doc]

Additional File 5.

**The top 500 differentially expressed genes in the transition from localized to metastatic prostate cancer - nMPC-MPC transition.**

| Gene_Symbol | Z-score | Direction | P-value |
| --- | --- | --- | --- |
| NR4A1 | -14.96 | Down | 6.73E-51 |
| MYH11 | -14.30 | Down | 1.02E-46 |
| IER2 | -14.20 | Down | 4.32E-46 |
| MEIS2 | -12.99 | Down | 7.28E-39 |
| EGR1 | -12.66 | Down | 5.11E-37 |
| CTGF | -12.59 | Down | 1.13E-36 |
| CYR61 | -12.32 | Down | 3.51E-35 |
| PCTK1 | 12.23 | Up | 1.652E-10 |
| SYNPO2 | -12.20 | Down | 1.55E-34 |
| TOP2A | 12.19 | Up | 1.657E-10 |
| MYLK | -11.93 | Down | 3.89E-33 |
| PAGE4 | -11.93 | Down | 4.10E-33 |
| ZMYND11 | -11.92 | Down | 4.87E-33 |
| IQGAP2 | -11.91 | Down | 5.15E-33 |
| CSRP1 | -11.74 | Down | 3.95E-32 |
| CALD1 | -11.72 | Down | 5.10E-32 |
| TCF21 | -11.65 | Down | 1.11E-31 |
| PTN | -11.49 | Down | 7.37E-31 |
| ATF3 | -11.38 | Down | 2.50E-30 |
| MYL9 | -11.37 | Down | 2.94E-30 |
| TPM2 | -11.31 | Down | 6.19E-30 |
| GOLGB1 | -11.28 | Down | 8.40E-30 |
| ZFP36 | -11.23 | Down | 1.44E-29 |
| SELE | -11.08 | Down | 8.14E-29 |
| PPP1R12B | -10.98 | Down | 2.26E-28 |
| BMPR1A | -10.94 | Down | 3.56E-28 |
| CNN1 | -10.93 | Down | 4.08E-28 |
| CCL2 | -10.93 | Down | 4.17E-28 |
| MGP | -10.89 | Down | 6.60E-28 |
| SRF | -10.72 | Down | 4.24E-27 |
| ZMYND8 | 10.61 | Up | 3.008E-29 |
| KRT15 | -10.50 | Down | 4.17E-26 |
| TAGLN | -10.48 | Down | 5.58E-26 |
| TPSAB1 | -10.43 | Down | 8.80E-26 |
| FHL1 | -10.43 | Down | 9.26E-26 |
| NR4A3 | -10.42 | Down | 1.03E-25 |
| FUCA1 | -10.40 | Down | 1.21E-25 |
| DIO2 | -10.27 | Down | 4.84E-25 |
| PCP4 | -10.25 | Down | 6.04E-25 |
| HLA-DRB1 | -10.05 | Down | 4.37E-24 |
| VCL | -10.02 | Down | 6.06E-24 |
| CTSO | -10.01 | Down | 6.85E-24 |
| CSNK1G2 | 9.97 | Up | 4.273E-29 |
| TMEM49 | -9.89 | Down | 2.41E-23 |
| SMAD4 | -9.88 | Down | 2.50E-23 |
| LMAN1 | -9.87 | Down | 2.81E-23 |
| FOSB | -9.84 | Down | 3.62E-23 |
| AGL | -9.82 | Down | 4.72E-23 |
| SPG20 | -9.82 | Down | 4.75E-23 |
| ABCC4 | -9.81 | Down | 5.21E-23 |
| PTPRK | -9.80 | Down | 5.80E-23 |
| DST | -9.78 | Down | 6.64E-23 |
| DUSP5 | -9.77 | Down | 7.40E-23 |
| ECM2 | -9.75 | Down | 9.47E-23 |
| PAM | -9.73 | Down | 1.07E-22 |
| DMXL1 | -9.70 | Down | 1.44E-22 |
| KRT5 | -9.68 | Down | 1.86E-22 |
| TIPARP | -9.67 | Down | 2.04E-22 |
| SSR3 | -9.64 | Down | 2.64E-22 |
| ID1 | -9.63 | Down | 2.96E-22 |
| BTG2 | -9.62 | Down | 3.17E-22 |
| PGK1 | 9.61 | Up | 4.318E-24 |
| SATB1 | -9.60 | Down | 3.83E-22 |
| UBE2J1 | -9.60 | Down | 4.15E-22 |
| TPM1 | -9.57 | Down | 5.36E-22 |
| MEIS1 | -9.56 | Down | 6.02E-22 |
| SFRS2B | -9.52 | Down | 8.50E-22 |
| CENPC1 | -9.46 | Down | 1.52E-21 |
| C18orf1 | -9.46 | Down | 1.53E-21 |
| FOXF1 | -9.45 | Down | 1.72E-21 |
| GADD45B | -9.44 | Down | 1.93E-21 |
| SMTN | -9.39 | Down | 2.92E-21 |
| SORBS2 | -9.35 | Down | 4.40E-21 |
| MITF | -9.32 | Down | 5.65E-21 |
| PPAP2B | -9.32 | Down | 5.82E-21 |
| SPARCL1 | -9.30 | Down | 6.87E-21 |
| TNFAIP3 | -9.28 | Down | 8.57E-21 |
| CGRRF1 | -9.27 | Down | 9.58E-21 |
| KCNMB1 | -9.25 | Down | 1.12E-20 |
| PDE4D | -9.20 | Down | 1.83E-20 |
| PJA2 | -9.19 | Down | 1.90E-20 |
| MIA3 | -9.19 | Down | 1.96E-20 |
| RPS23 | -9.19 | Down | 2.02E-20 |
| KLF4 | -9.15 | Down | 2.74E-20 |
| ST13 | -9.15 | Down | 2.76E-20 |
| SLC35A3 | -9.09 | Down | 4.89E-20 |
| KIFC1 | 9.08 | Up | 4.32E-22 |
| STC2 | 9.05 | Up | 6.065E-21 |
| CACNB2 | -9.05 | Down | 7.15E-20 |
| PYROXD1 | -9.02 | Down | 9.24E-20 |
| TMEM47 | -9.01 | Down | 1.06E-19 |
| PLN | -8.99 | Down | 1.18E-19 |
| TIA1 | -8.99 | Down | 1.19E-19 |
| AURKA | 8.98 | Up | 2.82E-19 |
| STIP1 | 8.96 | Up | 3.559E-19 |
| HLA-DPB1 | -8.95 | Down | 1.82E-19 |
| ACTA2 | -8.94 | Down | 2.01E-19 |
| MFAP4 | -8.93 | Down | 2.20E-19 |
| PLA2G2A | -8.91 | Down | 2.59E-19 |
| MATN2 | -8.90 | Down | 2.73E-19 |
| Stratagene lung | -8.85 | Down | 4.41E-19 |
| ITPR2 | -8.84 | Down | 4.94E-19 |
| NEO1 | -8.82 | Down | 5.88E-19 |
| UTRN | -8.81 | Down | 6.49E-19 |
| PDE8B | -8.76 | Down | 9.99E-19 |
| PPP1R12A | -8.73 | Down | 1.30E-18 |
| SLC15A2 | -8.72 | Down | 1.45E-18 |
| ZNF650 | -8.71 | Down | 1.46E-18 |
| NR4A2 | -8.71 | Down | 1.47E-18 |
| NUP210 | 8.70 | Up | 5.92E-19 |
| ALDH18A1 | -8.70 | Down | 1.61E-18 |
| DKFZP564O0823 | -8.70 | Down | 1.66E-18 |
| ZBTB10 | -8.69 | Down | 1.79E-18 |
| UAP1 | -8.66 | Down | 2.41E-18 |
| SON | -8.65 | Down | 2.48E-18 |
| CCNA2 | 8.65 | Up | 7.185E-19 |
| RARRES1 | -8.61 | Down | 3.59E-18 |
| JUN | -8.61 | Down | 3.69E-18 |
| SFRS5 | -8.53 | Down | 7.14E-18 |
| TM9SF3 | -8.53 | Down | 7.25E-18 |
| METAP2 | -8.53 | Down | 7.62E-18 |
| WSB2 | 8.51 | Up | 4.537E-18 |
| RLN1 | -8.51 | Down | 9.01E-18 |
| TPX2 | 8.51 | Up | 6.642E-18 |
| UBE2S | 8.50 | Up | 1.829E-17 |
| JUNB | -8.49 | Down | 1.01E-17 |
| FBLN1 | -8.45 | Down | 1.50E-17 |
| MAP9 | -8.43 | Down | 1.74E-17 |
| DYNC1I2 | -8.40 | Down | 2.16E-17 |
| DNAJC3 | -8.40 | Down | 2.27E-17 |
| FLJ39822 | -8.39 | Down | 2.48E-17 |
| ZNF536 | -8.37 | Down | 2.78E-17 |
| PUM2 | -8.37 | Down | 2.88E-17 |
| DDX39 | 8.36 | Up | 1.922E-17 |
| PALLD | -8.34 | Down | 3.80E-17 |
| MYBL2 | 8.34 | Up | 2.048E-17 |
| TFRC | 8.33 | Up | 5.427E-17 |
| SLC20A2 | -8.33 | Down | 4.01E-17 |
| ISL1 | -8.31 | Down | 4.79E-17 |
| FLNC | -8.29 | Down | 5.84E-17 |
| PBXIP1 | -8.28 | Down | 6.00E-17 |
| CDC6 | 8.26 | Up | 6.629E-17 |
| UFM1 | -8.25 | Down | 7.94E-17 |
| C14orf24 | -8.25 | Down | 8.03E-17 |
| IL1R1 | -8.23 | Down | 9.35E-17 |
| TCEAL1 | -8.23 | Down | 9.62E-17 |
| RPL10 | -8.23 | Down | 9.75E-17 |
| FLNA | -8.22 | Down | 9.77E-17 |
| EGR2 | -8.22 | Down | 1.03E-16 |
| C9orf61 | -8.22 | Down | 1.04E-16 |
| CDC42EP3 | -8.21 | Down | 1.07E-16 |
| ARMCX3 | -8.21 | Down | 1.07E-16 |
| ERGIC2 | -8.19 | Down | 1.30E-16 |
| SF1 | -8.19 | Down | 1.32E-16 |
| DKFZP586H2123 | -8.18 | Down | 1.37E-16 |
| AKT3 | -8.18 | Down | 1.40E-16 |
| DSTN | -8.16 | Down | 1.62E-16 |
| GHR | -8.16 | Down | 1.64E-16 |
| ALDH1A3 | -8.16 | Down | 1.70E-16 |
| CD69 | -8.14 | Down | 1.96E-16 |
| PTPRN2 | -8.13 | Down | 2.17E-16 |
| CREB3L1 | -8.12 | Down | 2.24E-16 |
| PRDM2 | -8.12 | Down | 2.25E-16 |
| FZD7 | -8.12 | Down | 2.35E-16 |
| PIGK | -8.11 | Down | 2.56E-16 |
| PKIG | -8.09 | Down | 3.04E-16 |
| BMI1 | -8.08 | Down | 3.15E-16 |
| NCKIPSD | -8.08 | Down | 3.37E-16 |
| FAM114A1 | -8.07 | Down | 3.58E-16 |
| CDKN3 | 8.06 | Up | 6.696E-17 |
| GULP1 | -8.06 | Down | 3.95E-16 |
| PTTG1 | 8.05 | Up | 1.14E-16 |
| ENC1 | -8.04 | Down | 4.59E-16 |
| AR | 8.03 | Up | 5.046E-16 |
| SLC30A7 | -8.02 | Down | 5.18E-16 |
| TMEM118 | 8.02 | Up | 5.238E-16 |
| SEMA3C | -8.01 | Down | 5.68E-16 |
| ARL6IP5 | -8.01 | Down | 5.69E-16 |
| BRD2 | 8.00 | Up | 5.945E-16 |
| NBL1 | -8.00 | Down | 6.44E-16 |
| RND3 | -7.99 | Down | 6.78E-16 |
| LOC171220 | -7.97 | Down | 7.78E-16 |
| PCM1 | -7.96 | Down | 8.56E-16 |
| RAP1A | -7.95 | Down | 8.97E-16 |
| SPOCK3 | -7.95 | Down | 9.10E-16 |
| SLC4A4 | -7.95 | Down | 9.68E-16 |
| AYTL2 | 7.94 | Up | 6.508E-16 |
| PTGS2 | -7.94 | Down | 1.02E-15 |
| NET1 | -7.93 | Down | 1.06E-15 |
| MKI67 | 7.92 | Up | 7.5E-16 |
| RAB6B | 7.92 | Up | 1.179E-15 |
| NBPF15 | -7.91 | Down | 1.33E-15 |
| KLF6 | -7.90 | Down | 1.44E-15 |
| GRB2 | 7.87 | Up | 1.385E-15 |
| TM9SF2 | -7.86 | Down | 1.93E-15 |
| SFRP1 | -7.85 | Down | 2.03E-15 |
| MMP7 | -7.84 | Down | 2.24E-15 |
| SLC12A2 | -7.82 | Down | 2.62E-15 |
| TIE1 | 7.81 | Up | 1.402E-15 |
| RPL5 | -7.80 | Down | 3.01E-15 |
| PIGB | -7.80 | Down | 3.02E-15 |
| OGN | -7.78 | Down | 3.69E-15 |
| MTMR6 | -7.76 | Down | 4.18E-15 |
| SLAIN2 | -7.73 | Down | 5.24E-15 |
| LOC440295 | -7.73 | Down | 5.48E-15 |
| EYA1 | -7.72 | Down | 5.60E-15 |
| PLAGL1 | -7.72 | Down | 5.75E-15 |
| RBPMS | -7.71 | Down | 6.23E-15 |
| PLK1 | 7.71 | Up | 2.009E-15 |
| CD36 | 7.69 | Up | 3.025E-15 |
| GSN | -7.67 | Down | 8.46E-15 |
| SUV420H1 | -7.65 | Down | 9.82E-15 |
| MPDZ | -7.65 | Down | 9.95E-15 |
| SLC2A3 | -7.65 | Down | 9.96E-15 |
| SPCS3 | -7.64 | Down | 1.05E-14 |
| ACTG2 | -7.63 | Down | 1.18E-14 |
| PIGN | -7.61 | Down | 1.39E-14 |
| RYBP | -7.60 | Down | 1.45E-14 |
| LGALS3 | -7.60 | Down | 1.48E-14 |
| PRKACB | -7.60 | Down | 1.52E-14 |
| SSB | -7.59 | Down | 1.59E-14 |
| SOD3 | -7.59 | Down | 1.62E-14 |
| TP63 | -7.58 | Down | 1.76E-14 |
| LTBP4 | -7.57 | Down | 1.92E-14 |
| KIAA0776 | -7.56 | Down | 2.01E-14 |
| TERF2IP | -7.56 | Down | 2.09E-14 |
| LYN | 7.55 | Up | 6.348E-15 |
| SAC3D1 | 7.55 | Up | 7.216E-15 |
| CNTN1 | -7.55 | Down | 2.19E-14 |
| COX7A1 | -7.55 | Down | 2.23E-14 |
| HLA-DQB1 | -7.55 | Down | 2.24E-14 |
| SLC35A1 | -7.54 | Down | 2.42E-14 |
| ID2 | -7.53 | Down | 2.53E-14 |
| NSUN7 | -7.53 | Down | 2.58E-14 |
| DDR2 | -7.52 | Down | 2.77E-14 |
| VDR | 7.50 | Up | 2.03E-14 |
| STX17 | -7.49 | Down | 3.57E-14 |
| LUM | -7.48 | Down | 3.60E-14 |
| DLG7 | 7.47 | Up | 2.033E-14 |
| CDC2 | 7.47 | Up | 2.867E-14 |
| RAB4A | -7.45 | Down | 4.78E-14 |
| KCNAB1 | -7.43 | Down | 5.30E-14 |
| SNAP23 | -7.43 | Down | 5.38E-14 |
| FOXM1 | 7.42 | Up | 3.534E-14 |
| TUBG1 | 7.42 | Up | 3.609E-14 |
| IDE | -7.42 | Down | 6.08E-14 |
| MXRA5 | -7.41 | Down | 6.25E-14 |
| CIRBP | -7.41 | Down | 6.37E-14 |
| SRI | -7.40 | Down | 6.66E-14 |
| PGM3 | -7.40 | Down | 6.94E-14 |
| SGMS1 | -7.38 | Down | 8.15E-14 |
| HSF1 | 7.37 | Up | 5.104E-14 |
| IQGAP1 | -7.35 | Down | 9.64E-14 |
| FNDC3A | -7.35 | Down | 9.82E-14 |
| SLC30A9 | -7.34 | Down | 1.04E-13 |
| EXTL2 | -7.34 | Down | 1.09E-13 |
| BAG1 | -7.34 | Down | 1.09E-13 |
| FRY | -7.33 | Down | 1.15E-13 |
| KIAA1450 | -7.32 | Down | 1.22E-13 |
| TNKS2 | -7.32 | Down | 1.25E-13 |
| TRIO | 7.32 | Up | 5.309E-14 |
| ABCC5 | 7.31 | Up | 7.543E-14 |
| SFRS7 | -7.30 | Down | 1.39E-13 |
| ANGPT1 | -7.29 | Down | 1.50E-13 |
| ELOVL7 | -7.29 | Down | 1.52E-13 |
| EGR3 | -7.29 | Down | 1.60E-13 |
| ANKRD17 | -7.28 | Down | 1.62E-13 |
| TSPYL1 | -7.28 | Down | 1.67E-13 |
| CAPZA2 | -7.27 | Down | 1.85E-13 |
| AUH | -7.26 | Down | 1.93E-13 |
| NT5C2 | -7.25 | Down | 2.06E-13 |
| EIF5 | 7.25 | Up | 1.124E-13 |
| ZFX | -7.25 | Down | 2.15E-13 |
| CUGBP1 | 7.24 | Up | 1.208E-13 |
| PRUNE2 | -7.23 | Down | 2.40E-13 |
| SLC35F5 | -7.22 | Down | 2.55E-13 |
| CD46 | -7.21 | Down | 2.74E-13 |
| DUSP1 | -7.20 | Down | 2.97E-13 |
| MAN2B2 | -7.20 | Down | 2.99E-13 |
| ELL2 | -7.19 | Down | 3.22E-13 |
| RNF141 | -7.19 | Down | 3.27E-13 |
| AVEN | -7.19 | Down | 3.32E-13 |
| NEDD4L | -7.18 | Down | 3.55E-13 |
| FMOD | -7.17 | Down | 3.84E-13 |
| MPZL2 | -7.16 | Down | 4.14E-13 |
| PPP1R2 | -7.13 | Down | 4.95E-13 |
| ALDH3A2 | -7.12 | Down | 5.24E-13 |
| PDLIM3 | -7.12 | Down | 5.45E-13 |
| CNTN3 | -7.12 | Down | 5.46E-13 |
| PCMTD1 | -7.12 | Down | 5.59E-13 |
| PELO | -7.10 | Down | 6.19E-13 |
| SSPN | -7.10 | Down | 6.35E-13 |
| APPBP2 | -7.08 | Down | 7.09E-13 |
| COL15A1 | -7.08 | Down | 7.12E-13 |
| RPL7A | -7.07 | Down | 7.48E-13 |
| FILIP1L | -7.07 | Down | 7.96E-13 |
| ELF1 | -7.06 | Down | 8.06E-13 |
| TUBG2 | 7.06 | Up | 1.838E-13 |
| IDH3A | -7.06 | Down | 8.21E-13 |
| REV1 | -7.06 | Down | 8.28E-13 |
| MSMB | -7.06 | Down | 8.39E-13 |
| RASA1 | -7.06 | Down | 8.58E-13 |
| ISLR | -7.06 | Down | 8.63E-13 |
| C9orf5 | -7.05 | Down | 8.84E-13 |
| FAAH | -7.05 | Down | 9.25E-13 |
| TMEM50B | -7.03 | Down | 1.01E-12 |
| GAPDH | 7.03 | Up | 1.93E-13 |
| RPL31 | -7.03 | Down | 1.04E-12 |
| HLA-DRA | -7.03 | Down | 1.05E-12 |
| PSAP | -7.02 | Down | 1.07E-12 |
| septin4 | 7.02 | Up | 7.057E-13 |
| TNFSF10 | -6.99 | Down | 1.33E-12 |
| C10orf72 | -6.99 | Down | 1.38E-12 |
| FYN | 6.99 | Up | 9.011E-13 |
| LEPR | -6.98 | Down | 1.49E-12 |
| TCTN3 | -6.97 | Down | 1.56E-12 |
| C10orf56 | -6.97 | Down | 1.59E-12 |
| OIT3 | 6.97 | Up | 9.38E-13 |
| GPD1L | -6.96 | Down | 1.73E-12 |
| PPP1CB | -6.96 | Down | 1.75E-12 |
| TMEM50A | -6.96 | Down | 1.75E-12 |
| TFPI | 6.95 | Up | 1.233E-12 |
| ASTN2 | -6.95 | Down | 1.87E-12 |
| MAP1LC3A | -6.95 | Down | 1.88E-12 |
| METAP1 | -6.94 | Down | 1.91E-12 |
| LHX2 | 6.94 | Up | 1.44E-12 |
| MOXD1 | -6.94 | Down | 1.97E-12 |
| THBS4 | -6.93 | Down | 2.11E-12 |
| ESR1 | -6.93 | Down | 2.11E-12 |
| IFNGR1 | -6.92 | Down | 2.33E-12 |
| ACYP2 | -6.91 | Down | 2.42E-12 |
| CNTNAP2 | -6.91 | Down | 2.42E-12 |
| CSMD1 | 6.91 | Up | 1.627E-12 |
| LAPTM4A | -6.90 | Down | 2.54E-12 |
| C12orf23 | -6.90 | Down | 2.58E-12 |
| RFC5 | 6.90 | Up | 1.713E-12 |
| SERPINB5 | -6.88 | Down | 2.97E-12 |
| MED21 | -6.88 | Down | 3.08E-12 |
| SORL1 | -6.88 | Down | 3.09E-12 |
| RBP5 | 6.87 | Up | 2.197E-12 |
| UBE2G2 | 6.87 | Up | 2.316E-12 |
| NT5DC1 | -6.87 | Down | 3.20E-12 |
| CX3CL1 | -6.87 | Down | 3.32E-12 |
| COL4A6 | -6.86 | Down | 3.52E-12 |
| KIAA0101 | 6.86 | Up | 2.812E-12 |
| SNAI1 | 6.84 | Up | 2.882E-12 |
| ACPP | -6.84 | Down | 3.86E-12 |
| STXBP3 | -6.84 | Down | 3.95E-12 |
| RLN2 | -6.84 | Down | 4.05E-12 |
| GSTM1 | -6.83 | Down | 4.17E-12 |
| GOLM1 | -6.83 | Down | 4.18E-12 |
| PDIA3 | -6.83 | Down | 4.32E-12 |
| GLOD4 | -6.82 | Down | 4.71E-12 |
| GLUD1 | -6.81 | Down | 4.87E-12 |
| PIM1 | -6.81 | Down | 4.90E-12 |
| CREM | -6.80 | Down | 5.28E-12 |
| DACH1 | -6.79 | Down | 5.50E-12 |
| SLC2A10 | -6.79 | Down | 5.61E-12 |
| PVRL3 | -6.78 | Down | 5.87E-12 |
| C17orf48 | -6.78 | Down | 5.88E-12 |
| SMYD2 | 6.78 | Up | 3.2E-12 |
| ILK | -6.78 | Down | 6.21E-12 |
| DDIT4 | 6.77 | Up | 3.501E-12 |
| MAST1 | 6.77 | Up | 5.738E-12 |
| MAP3K9 | 6.77 | Up | 5.891E-12 |
| MLXIPL | 6.76 | Up | 6.001E-12 |
| ZBTB20 | -6.76 | Down | 6.72E-12 |
| ZRANB2 | -6.76 | Down | 7.05E-12 |
| SPOP | -6.75 | Down | 7.17E-12 |
| PDK3 | 6.75 | Up | 6.022E-12 |
| P2RY5 | -6.75 | Down | 7.38E-12 |
| NEK1 | -6.75 | Down | 7.39E-12 |
| FLJ33996 | 6.74 | Up | 6.284E-12 |
| DPF3 | 6.74 | Up | 6.814E-12 |
| GRAMD1B | 6.73 | Up | 7.318E-12 |
| MCL1 | -6.73 | Down | 8.41E-12 |
| ITM2B | -6.72 | Down | 9.11E-12 |
| PLXND1 | 6.71 | Up | 7.473E-12 |
| AK1 | 6.69 | Up | 7.823E-12 |
| PTPN13 | -6.69 | Down | 1.10E-11 |
| SRRM1 | -6.68 | Down | 1.21E-11 |
| ZNF451 | -6.68 | Down | 1.22E-11 |
| OSR2 | -6.67 | Down | 1.24E-11 |
| KIF13B | -6.67 | Down | 1.29E-11 |
| LOC157562 | 6.67 | Up | 9.374E-12 |
| KCNAB2 | 6.66 | Up | 1.051E-11 |
| DDX3Y | -6.66 | Down | 1.40E-11 |
| TARBP1 | -6.65 | Down | 1.44E-11 |
| EPS15 | -6.65 | Down | 1.45E-11 |
| EFNB1 | 6.65 | Up | 1.264E-11 |
| RANBP2 | -6.64 | Down | 1.55E-11 |
| RNF13 | -6.64 | Down | 1.58E-11 |
| PKN2 | -6.64 | Down | 1.58E-11 |
| PINK1 | -6.63 | Down | 1.65E-11 |
| SLC39A6 | -6.63 | Down | 1.68E-11 |
| RPA3 | 6.63 | Up | 1.338E-11 |
| DDHD2 | -6.63 | Down | 1.74E-11 |
| CETP | 6.62 | Up | 1.486E-11 |
| SEC14L1 | 6.62 | Up | 1.717E-11 |
| AFAP1 | -6.62 | Down | 1.82E-11 |
| AMD1 | -6.62 | Down | 1.83E-11 |
| DHX34 | 6.62 | Up | 1.8E-11 |
| RBM26 | -6.61 | Down | 1.88E-11 |
| ZNF468 | -6.60 | Down | 2.10E-11 |
| ARHGEF10 | -6.59 | Down | 2.13E-11 |
| C10orf118 | -6.59 | Down | 2.26E-11 |
| ASAH1 | -6.58 | Down | 2.34E-11 |
| KCTD3 | -6.58 | Down | 2.36E-11 |
| COCH | -6.58 | Down | 2.36E-11 |
| SH3BGR | -6.58 | Down | 2.41E-11 |
| CCNC | -6.57 | Down | 2.48E-11 |
| JUND | -6.57 | Down | 2.50E-11 |
| RP11-50D16.3 | -6.57 | Down | 2.51E-11 |
| MAP7 | -6.56 | Down | 2.65E-11 |
| TMF1 | -6.56 | Down | 2.67E-11 |
| TMEM168 | -6.56 | Down | 2.67E-11 |
| PTGER2 | -6.56 | Down | 2.73E-11 |
| ERCC5 | -6.56 | Down | 2.74E-11 |
| TMED10 | -6.56 | Down | 2.74E-11 |
| PRC1 | 6.55 | Up | 1.813E-11 |
| EIF3A | -6.54 | Down | 2.98E-11 |
| BOLA2 | 6.54 | Up | 1.86E-11 |
| RPLP1 | -6.54 | Down | 3.07E-11 |
| MFGE8 | -6.54 | Down | 3.11E-11 |
| GDI2 | -6.53 | Down | 3.33E-11 |
| MGC16121 | 6.53 | Up | 2.991E-11 |
| Gessler Wilms t | -6.51 | Down | 3.71E-11 |
| MTA1 | 6.51 | Up | 3.133E-11 |
| SCCPDH | -6.51 | Down | 3.79E-11 |
| RAB27B | -6.51 | Down | 3.79E-11 |
| LMNB2 | 6.49 | Up | 3.539E-11 |
| SMARCA2 | -6.49 | Down | 4.033E-11 |
| RNF103 | -6.49 | Down | 4.037E-11 |
| MSTP9 | -6.49 | Down | 4.699E-11 |
| SCRIB | 6.48 | Up | 3.977E-11 |
| CDC42BPA | -6.47 | Down | 4.722E-11 |
| TCF12 | -6.47 | Down | 4.771E-11 |
| DPP4 | -6.47 | Down | 5.2E-11 |
| PEG10 | 6.47 | Up | 4.688E-11 |
| CXADR | -6.46 | Down | 5.355E-11 |
| MICAL3 | 6.46 | Up | 5.052E-11 |
| FSCN1 | 6.46 | Up | 5.552E-11 |
| GOLPH3 | -6.46 | Down | 5.445E-11 |
| GARNL1 | -6.46 | Down | 5.567E-11 |
| EIF4G1 | 6.45 | Up | 5.661E-11 |
| KCTD5 | 6.44 | Up | 5.748E-11 |
| C10orf116 | -6.44 | Down | 5.839E-11 |
| MLLT4 | -6.43 | Down | 5.876E-11 |
| ALDOA | 6.43 | Up | 6.266E-11 |
| PARVB | 6.43 | Up | 6.789E-11 |
| VAMP3 | -6.42 | Down | 6.857E-11 |
| SH3YL1 | -6.42 | Down | 6.988E-11 |
| EZH2 | 6.41 | Up | 7.046E-11 |
| ZNF185 | -6.41 | Down | 7.485E-11 |
| RUFY3 | -6.41 | Down | 7.869E-11 |
| RPL35A | -6.41 | Down | 8.213E-11 |
| UBE2C | 6.40 | Up | 7.088E-11 |
| SOAT1 | -6.39 | Down | 8.376E-11 |
| HLA-DQA1 | -6.39 | Down | 8.494E-11 |
| CRABP1 | 6.39 | Up | 8.059E-11 |
| HOXA4 | 6.39 | Up | 8.913E-11 |
| TICAM2 | -6.39 | Down | 9.309E-11 |
| AKAP11 | -6.39 | Down | 9.483E-11 |
| LOH11CR2A | -6.39 | Down | 9.741E-11 |
| KIAA1109 | -6.38 | Down | 9.767E-11 |
| DIXDC1 | -6.38 | Down | 9.775E-11 |
| NDUFS4 | -6.38 | Down | 9.995E-11 |
| PLA2G5 | -6.38 | Down | 1.003E-10 |
| BTF3 | -6.38 | Down | 1.012E-10 |
| PLP1 | -6.37 | Down | 1.04E-10 |
| SMAD2 | -6.37 | Down | 1.066E-10 |
| KDELR3 | -6.36 | Down | 1.079E-10 |
| TCF3 | 6.36 | Up | 9.535E-11 |
| ATXN10 | -6.35 | Down | 1.13E-10 |
| TLOC1 | -6.35 | Down | 1.158E-10 |
| LMOD1 | -6.35 | Down | 1.247E-10 |
| PRKAR1A | -6.35 | Down | 1.248E-10 |
| HSP90AA1 | 6.35 | Up | 9.552E-11 |
| CCDC47 | -6.35 | Down | 1.269E-10 |
| DHRS3 | -6.35 | Down | 1.275E-10 |
| C10orf137 | -6.35 | Down | 1.287E-10 |
| LRPAP1 | -6.34 | Down | 1.293E-10 |
| TLK1 | -6.34 | Down | 1.321E-10 |
| KLF9 | -6.34 | Down | 1.36E-10 |
| OAT | -6.34 | Down | 1.385E-10 |
| PSMD5 | -6.34 | Down | 1.398E-10 |
| NF2 | 6.33 | Up | 1.171E-10 |
| ZHX2 | -6.33 | Down | 1.409E-10 |
| THBS2 | 6.33 | Up | 1.281E-10 |
| BUB1 | 6.33 | Up | 1.435E-10 |
| FOXK2 | 6.33 | Up | 1.436E-10 |
| DDX12 | 6.33 | Up | 1.447E-10 |
| HOXC6 | 6.32 | Up | 1.486E-10 |
| DIS3 | -6.32 | Down | 1.411E-10 |
| ARMC1 | -6.32 | Down | 1.436E-10 |
| MON1B | -6.32 | Down | 1.617E-10 |
| CCBL2 | -6.32 | Down | 1.629E-10 |
| ACSM3 | -6.32 | Down | 1.64E-10 |
| ACOT12 | 6.31 | Up | 1.49E-10 |
| SH3GL1 | 6.31 | Up | 1.592E-10 |
