# Supplementary material for: Candidate pathways and genes for prostate cancer: a meta-analysis of gene expression data
Source: BMC Med Genomics. 2009 Aug 4;2:48. doi: 10.1186/1755-8794-2-48 (PMC2731785; doi:10.1186/1755-8794-2-48)
Supplement: Additional file 7 — List of genes for Table 3 "Canonical significance in nonmetastatic prostate cancer to metastatic prostate cancer (nMPC-MPC) transition pathways identified by Ingenuity software". The data provided represent the list of genes for Table 3. [file 1755-8794-2-48-S7.doc]

Additional File 9.

**List of genes for the Table 3 “Canonical significance in nonmetastatic prostate cancer to metastatic prostate cancer (nMPC–MPC) transition pathways identified by Ingenuity software”.**

| Pathway | Genes |
| --- | --- |
| Tight Junction Signaling | PRKACB, HSF1, ACTA2 (includes EG:59), PVRL3, MLLT4, MYH11, MYLK, MYL9 (includes EG:10398), MPDZ, JUN, AKT3, VCL, ACTG2, PRKAR1A |
| IGF-1 Signaling | PRKACB, CTGF, JUN, GRB2, SRF, AKT3, CYR61, RASA1, PRKAR1A |
| Integrin Signaling | FYN, GRB2, ACTA2 (includes EG:59), ILK, PPP1CB, RAP1A, MYLK, PARVB, RND3, PPP1R12B, AKT3, PPP1R12A, VCL, ACTG2 |
| ERK/MAPK Signaling | PRKACB, FYN, GRB2, SRF, PPP1CB, PLA2G2A, RAP1A, ELF1, DUSP1, PLA2G5, PPP1R12A, ESR1, PRKAR1A |
| Regulation of Actin-based Motility by Rho | MYLK, RND3, ACTA2 (includes EG:59), PPP1R12B, PPP1CB, PPP1R12A, ACTG2, GSN |
| Hepatic Fibrosis / Hepatic Stellate Cell Activation | MYL9 (includes EG:10398), SMAD2, CTGF, CCL2, LEPR, ACTA2 (includes EG:59), SMAD4, IFNGR1, MYH11, IL1R1 |
| Cardiac beta²-adrenergic Signaling | PRKACB, PLN, PPP1CB, PPP1R12A, PDE8B, PDE4D, PKIG, PRKAR1A, AKAP11 |
| EGF Signaling | JUN, ITPR2, GRB2, SRF, RASA1 |
| Actin Cytoskeleton Signaling | GRB2, ACTA2 (includes EG:59), PPP1CB, MYH11, IQGAP1 (includes EG:8826), GSN, MYL9 (includes EG:10398), MYLK, IQGAP2, PPP1R12B, PPP1R12A, VCL, ACTG2 |
| PPARα/RXRα Activation | PRKACB, SMAD2, GHR, JUN, PDIA3, GRB2, CD36, HSP90AA1, SMAD4, IL1R1, PRKAR1A |
| IL-4 Signaling | HLA-DQB1, GRB2, HLA-DRA, HLA-DQA1, AKT3, HLA-DRB1 |
| Nitric Oxide Signaling in the Cardiovascular System | PRKACB, PLN, ITPR2, HSP90AA1, AKT3, PRKAR1A |
| Hypoxia Signaling in the Cardiovascular System | UBE2G2, JUN, HSP90AA1, UBE2S, UBE2J1, UBE2C |
| Antigen Presentation Pathway | HLA-DRA, HLA-DQA1, HLA-DRB1, HLA-DPB1 |
| Glucocorticoid Receptor Signaling | PRKACB, SMAD2, SELE, GRB2, JUN, AR, CCL2, DUSP1, SMARCA2, BAG1, SMAD4, HSP90AA1, AKT3, ESR1 |
| Glycolysis/Gluconeogenesis | PGK1, PGM3, ALDH1A3, ALDH3A2, GAPDH (includes EG:2597), ALDOA, ACYP2 |
| TGF-beta Signaling | SMAD2, JUN, BMPR1A, GRB2, SMAD4, VDR |
