# Supplementary material for: Candidate pathways and genes for prostate cancer: a meta-analysis of gene expression data
Source: BMC Med Genomics. 2009 Aug 4;2:48. doi: 10.1186/1755-8794-2-48 (PMC2731785; doi:10.1186/1755-8794-2-48)
Supplement: Additional file 8 — List of genes for Table 4 "Canonical pathways identified in the combined analysis of genes differentially expressed between the transitions from normal prostate to primary nonmetastatic prostate cancer (NP-nMPC) and primary nonmetatatic to metastatic prostate cancer (nMPC-MPC)". The data provided represent the list of genes for Table 4. [file 1755-8794-2-48-S8.doc]

Additional File 10.

**List of genes for the Table 4 “Canonical pathways identified in the combined analysis of genes differentially expressed between the transitions from normal prostate to primary nonmetastatic prostate cancer (NP–nMPC) and primary nonmetatatic to metastatic prostate cancer (nMPC–MPC)”.**

| Pathway | Genes |
| --- | --- |
| Integrin Signaling | PARVA, ITGA2, ITGA8 (includes EG:8516), ACTA2 (includes EG:59), ILK, TSPAN2, ITGB8, RAP1A, MYLK, DOCK1, RND3, RHOA, CAV1, PPP1R12B, PPP1R12A, AKT3, ACTG2, VCL, ACTN1 |
| Hepatic Fibrosis / Hepatic Stellate Cell Activation | CTGF, LEPR, FGFR1, ACTA2 (includes EG:59), SMAD7, FGFR2, MYH11, IL1R1, MYL9 (includes EG:10398), CCL2, IGF1, SMAD4, EDNRA |
| Actin Cytoskeleton Signaling | ITGA2, ACTA2 (includes EG:59), MYH11, GSN, IQGAP1 (includes EG:8826), MYLK, ROCK2, MYL9 (includes EG:10398), DOCK1, CYFIP2, CFL2, RHOA, PPP1R12B, PPP1R12A, VCL, ACTG2, ACTN1 |
| Tight Junction Signaling | ACTA2 (includes EG:59), MYH11, MYLK, MYL9 (includes EG:10398), MPDZ, PPP2CB, JUN, JAM3, RHOA, AKT3, VCL, ACTG2, MAGI2, PRKAR1A |
| Chemokine Signaling | ROCK2, CAMK2D, JUN, CCL2, RHOA, PPP1R12B, PPP1R12A, CALM1, CAMK2G |
| Calcium Signaling | TPM1, TP63, ACTA2 (includes EG:59), RCAN2, TPM2, MYH11, ITPR1, RAP1A, MYL9 (includes EG:10398), CAMK2D, ATP2B4, CAMK2G, PRKAR1A, CALM1 |
| IGF-1 Signaling | CTGF, JUN, IGF1, IRS1, SRF, AKT3, CYR61, SFN, PRKAR1A |
| Aryl Hydrocarbon Receptor Signaling | CCNA2, GSTM2, CCND2, JUN, GSTM5, ALDH3A2, NFIA, ALDH1A2, RARB, NFKB1, NFE2L2 |
| Regulation of Actin-based Motility by Rho | MYLK, RND3, RHOA, ACTA2 (includes EG:59), PPP1R12B, PPP1R12A, ACTG2, GSN |
| Nitric Oxide Signaling in the Cardiovascular System | PLN, CAV1, AKT3, SLC7A1, ITPR1, PRKAR1A, CALM1 |
| Beta-alanine Metabolism | AOC3, SRM, DPYD, DPYSL3, ALDH3A2, ALDH1A2 |
| Wnt/β-catenin Signaling | SOX4, PPP2CB, GJA1, TCF4, CSNK1G2, DKK3, RARB, ILK, AKT3, TCF3, FZD7 |
| VEGF Signaling | ROCK2, ACTA2 (includes EG:59), AKT3, VCL, ACTG2, SFN, ACTN1 |
| p53 Signaling | PLAGL1, TP63, CCND2, JUN, AKT3, SERPINB5, SFN |
| NRF2-mediated Oxidative Stress Response | GSTM2, JUN, GSTM5, STIP1, MAF, ACTA2 (includes EG:59), HSPB8, DNAJC10, ACTG2, NFE2L2, SOD3 |
| Arginine and Proline Metabolism | AOC3, SRM, P4HA1, OAT, ALDH3A2, ALDH1A2 |
| Cardiac β-adrenergic Signaling | PPP2CB, PLN, PPP1R12A, PDE8B, PDE4D, PKIG, PRKAR1A, AKAP11 |
| cAMP-mediated Signaling | CREM, CAMK2D, PDE8B, PDE4D, RAP1A, PKIG, PRKAR1A, CALM1, AKAP11, CAMK2G |
| Cell Cycle: G2/M DNA Damage Checkpoint Regulation | TOP2A, SFN, SKP1, CDC2 |
