# Supplementary material for: Candidate pathways and genes for prostate cancer: a meta-analysis of gene expression data
Source: BMC Med Genomics. 2009 Aug 4;2:48. doi: 10.1186/1755-8794-2-48 (PMC2731785; doi:10.1186/1755-8794-2-48)
Supplement: Additional file 9 — Changes in gene expression of different cell adhesion molecule in NP-nMPC transition. The data provided represent changes in gene expression of different cell adhesion molecule in NP-nMPC transition. [file 1755-8794-2-48-S9.doc]

Additional File 6.

**Changes in gene expression of different cell adhesion molecule in NP-nMPC transition.**

| Gene | Type | Direction | Z-score | P-value |
| --- | --- | --- | --- | --- |
| PCDH7 | cadherin | Down | 6.918659978 | 4.55936E-12 |
| CTNND2 | cadherin | Up | 6.256674757 | 5.5936E-11 |
| PCDHAC2 | cadherin | Up | 5.94890466 | 2E-09 |
| PCDHGC3 | cadherin | Up | 5.347076208 | 9E-08 |
| PCDHB2 | cadherin | Up | 4.748857859 | 2.046E-06 |
| DSC2 | cadherin | Up | 4.583037836 | 4.582E-06 |
| CDH19 | cadherin | Down | 4.318378468 | 1.5718E-05 |
| PCDH1 | cadherin | Up | 4.182418284 | 2.8842E-05 |
| CDH12 | cadherin | Up | 4.172324589 | 3.015E-05 |
| PCDHB10 | cadherin | Up | 4.02020452 | 5.8148E-05 |
| PCDHA12 | cadherin | Up | 3.961052458 | 7.462E-05 |
| DSC3 | cadherin | Down | 3.705251159 | 0.000211182 |
| CELSR2 | cadherin | Down | 3.639895157 | 0.00027275 |
| CDH3 | cadherin | Down | 3.538390717 | 0.000402574 |
| CELSR3 | cadherin | Up | 3.50369017 | 0.000458858 |
| PCDH18 | cadherin | Down | 3.493277569 | 0.00047713 |
| DSG2 | cadherin | Up | 3.448474596 | 0.000563762 |
| PCDHB5 | cadherin | Up | 3.284131142 | 0.001022974 |
| PCDHB14 | cadherin | Up | 3.275616116 | 0.001054318 |
| CDH1 | cadherin | Up | 3.255353253 | 0.001132514 |
| CDH13 | cadherin | Up | 3.226515772 | 0.001253074 |
| DCHS1 | cadherin | Down | 3.089369335 | 0.00200582 |
| PCDHB8 | cadherin | Up | 3.04747977 | 0.00230769 |
| PCDHB16 | cadherin | Up | 2.927982396 | 0.003411694 |
| JUP | cadherin | Up | 2.903086749 | 0.003695042 |
| PCDH20 | cadherin | Up | 2.826935931 | 0.004699572 |
| PCDHGA8 | cadherin | Up | 2.797270241 | 0.00515364 |
| PCDHA6 | cadherin | Up | 2.706536945 | 0.006798902 |
| CDH11 | cadherin | Up | 2.699756044 | 0.006939034 |
| CDH26 | cadherin | Down | 2.591180124 | 0.009564742 |
| PCDHGA10 | cadherin | Up | 2.425699537 | 0.015278914 |
| PCDHA9 | cadherin | Up | 2.411335727 | 0.01589421 |
| PCDHGA1 | cadherin | Up | 2.323644377 | 0.020144562 |
| CDH6 | cadherin | Down | 2.198867252 | 0.027887362 |
| CDH15 | cadherin | Down | 2.171879062 | 0.029864786 |
| CDH5 | cadherin | Down | 2.110069291 | 0.034852388 |
| CTNNB1 | cadherin | Down | 2.043381545 | 0.041014682 |
| PCDH8 | cadherin | Up | 1.978656333 | 0.047854712 |
| PCDH9 | cadherin | Down | 1.909890427 | 0.056147322 |
| CDON | cadherin | Up | 1.901444297 | 0.057243842 |
| PCDHB9 | cadherin | Down | 1.898251217 | 0.057662996 |
| PCDHGA3 | cadherin | Up | 1.896832366 | 0.057850066 |
| PCDHGC5 | cadherin | Down | 1.778541259 | 0.075315002 |
| CDH23 | cadherin | Down | 1.744114263 | 0.081139168 |
| PCDHGA9 | cadherin | Down | 1.702325911 | 0.08869429 |
| DCHS2 | cadherin | Down | 1.693289622 | 0.090400352 |
| PCDHB12 | cadherin | Up | 1.620491535 | 0.105126732 |
| PCDH17 | cadherin | Up | 1.556608675 | 0.119563422 |
| DSG4 | cadherin | Down | 1.552822199 | 0.120465618 |
| PCDHA2 | cadherin | Up | 1.550590111 | 0.120999944 |
| PCDH11Y | cadherin | Down | 1.538216274 | 0.123995744 |
| PCDHB13 | cadherin | Up | 1.511486501 | 0.130664546 |
| PCDHB4 | cadherin | Up | 1.498951787 | 0.13388614 |
| PCDHB11 | cadherin | Up | 1.497351496 | 0.134301822 |
| PCDH12 | cadherin | Down | 1.470671887 | 0.141379872 |
| CDH18 | cadherin | Down | 1.462971449 | 0.143475192 |
| CDH4 | cadherin | Up | 1.448213431 | 0.14755737 |
| CELSR1 | cadherin | Down | 1.435208563 | 0.151227674 |
| PCDHB7 | cadherin | Up | 1.388178896 | 0.165082576 |
| DSG3 | cadherin | Down | 1.367672379 | 0.17141465 |
| PCDHGB4 | cadherin | Down | 1.313053845 | 0.189164804 |
| PCDH10 | cadherin | Down | 1.218186315 | 0.223153178 |
| PCDHGB5 | cadherin | Up | 1.21134951 | 0.225761482 |
| PCDH11X | cadherin | Up | 1.150302402 | 0.250019342 |
| PCDHB18 | cadherin | Up | 1.134935107 | 0.256402508 |
| PCDHA10 | cadherin | Up | 1.131035317 | 0.258040226 |
| PCDHB1 | cadherin | Up | 1.092450585 | 0.274635098 |
| CDH2 | cadherin | Down | 1.071212339 | 0.284073964 |
| CDH24 | cadherin | Down | 1.068247344 | 0.285408954 |
| PCDH21 | cadherin | Down | 1.057267659 | 0.290389448 |
| PCDHGB7 | cadherin | Down | 1.004339801 | 0.315214856 |
| CDH9 | cadherin | Up | 0.987492605 | 0.323401206 |
| CDH20 | cadherin | Down | 0.946458823 | 0.343914616 |
| PCDHB17 | cadherin | Up | 0.942903641 | 0.345730192 |
| PCDH19 | cadherin | Up | 0.933099868 | 0.350768408 |
| PCDHGC4 | cadherin | Down | 0.801276174 | 0.422971782 |
| CDH16 | cadherin | Down | 0.793369095 | 0.427562812 |
| PCDHB19P | cadherin | Down | 0.784425456 | 0.432790506 |
| CDH17 | cadherin | Down | 0.77913665 | 0.435899224 |
| PCDHB3 | cadherin | Up | 0.732085497 | 0.464116386 |
| PCDHA5 | cadherin | Up | 0.67554811 | 0.499327596 |
| CDH8 | cadherin | Down | 0.615166518 | 0.538444756 |
| PCDHB6 | cadherin | Up | 0.52164458 | 0.601917818 |
| PCDHA3 | cadherin | Up | 0.468470286 | 0.639448316 |
| PCDHB15 | cadherin | Up | 0.440925673 | 0.659266808 |
| CDH7 | cadherin | Up | 0.390983097 | 0.69580973 |
| DSG1 | cadherin | Down | 0.359895015 | 0.718925646 |
| PCDH15 | cadherin | Down | 0.335238435 | 0.737445232 |
| PCDHGA11 | cadherin | Down | 0.290568213 | 0.771381574 |
| CDH22 | cadherin | Up | 0.286685543 | 0.774353108 |
| PCDHGA12 | cadherin | Up | 0.122994734 | 0.902111268 |
| DSC1 | cadherin | Up | 0.073156062 | 0.94168193 |
| CDH10 | cadherin | Up | 0.016914496 | 0.986504828 |
| PCDHAC1 | cadherin | Up | 0.015792707 | 0.987399766 |
| CTNND1 | cadherin | Up | 0.015647325 | 0.98751575 |
| NCAM1 | ICAM_TCAM | Down | 7.74817033 | 9.3226E-15 |
| ICAM3 | ICAM_TCAM | Up | 7.67046811 | 7.3226E-15 |
| ICAM2 | ICAM_TCAM | Down | 3.20447734 | 0.00135308 |
| ICAM4 | ICAM_TCAM | Down | 2.817970686 | 0.004832822 |
| ICAM1 | ICAM_TCAM | Down | 2.163456411 | 0.030506092 |
| ICAM5 | ICAM_TCAM | Down | 1.656816907 | 0.097556498 |
| CLEC4M | ICAM_TCAM | Down | 0.617782927 | 0.53671844 |
| CAV1 | integrin | Down | 11.96401215 | 5.48464E-33 |
| ITGA2 | integrin | Down | 10.33194836 | 5.05238E-25 |
| ITGA3 | integrin | Down | 8.150246762 | 3.63182E-16 |
| ITGB4 | integrin | Down | 7.267457542 | 3.66316E-13 |
| ITGA1 | integrin | Down | 6.543747012 | 5.99962E-11 |
| ITGA9 | integrin | Down | 6.530958615 | 6.535E-11 |
| ITGA7 | integrin | Down | 5.91747343 | 3.26924E-09 |
| ITGA5 | integrin | Down | 5.458526227 | 4.80102E-08 |
| ITGA8 | integrin | Down | 5.45175484 | 4.98752E-08 |
| ITGB1BP2 | integrin | Down | 4.448513699 | 8.64666E-06 |
| ITGB6 | integrin | Down | 4.062459313 | 4.85584E-05 |
| ITGB1 | integrin | Down | 3.974037675 | 7.06644E-05 |
| ILK | integrin | Down | 3.880321729 | 0.000104318 |
| CELSR2 | integrin | Down | 3.639895157 | 0.00027275 |
| CNTNAP2 | integrin | Up | 3.571957429 | 0.000354324 |
| CELSR3 | integrin | Up | 3.50369017 | 0.000458858 |
| CNTNAP1 | integrin | Down | 3.315064633 | 0.00091622 |
| NRXN1 | integrin | Down | 3.065401932 | 0.002173776 |
| ITGAE | integrin | Down | 2.995563119 | 0.002739386 |
| CNTNAP3B | integrin | Up | 2.962716523 | 0.003049372 |
| ITFG2 | integrin | Down | 2.920816354 | 0.003491156 |
| ICAM4 | integrin | Down | 2.817970686 | 0.004832822 |
| ITGAX | integrin | Up | 2.621626828 | 0.00875112 |
| ICAM1 | integrin | Down | 2.163456411 | 0.030506092 |
| ITGB3BP | integrin | Up | 1.901678802 | 0.057213158 |
| ITGB1BP3 | integrin | Down | 1.819965277 | 0.068764292 |
| ITGB3 | integrin | Down | 1.747135058 | 0.080613914 |
| NRXN3 | integrin | Down | 1.561421344 | 0.11842437 |
| ILKAP | integrin | Down | 1.524724108 | 0.127327926 |
| CDH18 | integrin | Down | 1.462971449 | 0.143475192 |
| CELSR1 | integrin | Down | 1.435208563 | 0.151227674 |
| NRXN2 | integrin | Down | 1.233718095 | 0.217307968 |
| ITGA4 | integrin | Up | 1.229050185 | 0.21905299 |
| ITGB1BP1 | integrin | Down | 1.182552866 | 0.236986402 |
| ITGAD | integrin | Up | 1.151834818 | 0.249388958 |
| CNTNAP3 | integrin | Up | 1.056651883 | 0.290670492 |
| CNTNAP5 | integrin | Up | 1.010431903 | 0.31228841 |
| ITGA2B | integrin | Up | 0.849836623 | 0.395415926 |
| ITGAV | integrin | Up | 0.833370112 | 0.404636026 |
| CDH16 | integrin | Down | 0.793369095 | 0.427562812 |
| CDH17 | integrin | Down | 0.77913665 | 0.435899224 |
| CNTNAP4 | integrin | Up | 0.702111711 | 0.4826095 |
| ITGA11 | integrin | Down | 0.698343258 | 0.484962552 |
| ITGAM | integrin | Up | 0.691845022 | 0.48903466 |
| ITGB2 | integrin | Down | 0.680639562 | 0.496099588 |
| ITGA10 | integrin | Down | 0.617157254 | 0.537131008 |
| ITGB5 | integrin | Up | 0.585426069 | 0.558261264 |
| ITGAL | integrin | Up | 0.393793106 | 0.693733794 |
| ITGB7 | integrin | Down | 0.358674963 | 0.71983826 |
| ITGA6 | integrin | Down | 0.160024834 | 0.872861512 |
| CDH10 | integrin | Up | 0.016914496 | 0.986504828 |
| VCL | selectin | Down | 12.93377106 | 2.90226E-38 |
| CD44 | selectin | Down | 4.340557706 | 1.42122E-05 |
| SELENBP1 | selectin | Down | 4.204706783 | 0.000026142 |
| SELPLG | selectin | Down | 1.44384051 | 0.148783844 |
| SELP | selectin | Down | 1.167074521 | 0.243180276 |
| SELE | selectin | Down | 0.79167357 | 0.428551038 |
| ACTN2 | selectin | Up | 0.292175368 | 0.770152544 |
| SELL | selectin | Up | 0.076331959 | 0.939155 |
| GJA1 | tight_junction | Down | 11.38165755 | 5.16094E-30 |
| OCLN | tight_junction | Up | 8.898251201 | 1.09E-25 |
| CLDN8 | tight_junction | Up | 8.489438612 | 6.09E-25 |
| JAM3 | tight_junction | Down | 8.425612731 | 3.58868E-17 |
| GJC1 | tight_junction | Down | 7.19471972 | 2.5892E-13 |
| TJP1 | tight_junction | Up | 6.95345098 | 2.5892E-11 |
| CLDN7 | tight_junction | Up | 6.744164259 | 6.25892E-11 |
| CLDN4 | tight_junction | Up | 5.338983069 | 9.4E-08 |
| CLDN3 | tight_junction | Up | 5.266652196 | 1.38E-07 |
| CDH19 | tight_junction | Down | 4.318378468 | 1.5718E-05 |
| CDH12 | tight_junction | Up | 4.172324589 | 3.015E-05 |
| CTNNA1 | tight_junction | Down | 4.047882209 | 5.16832E-05 |
| TJP3 | tight_junction | Down | 3.930221988 | 8.48674E-05 |
| CDH1 | tight_junction | Up | 3.255353253 | 0.001132514 |
| CDH13 | tight_junction | Up | 3.226515772 | 0.001253074 |
| CLDN10 | tight_junction | Up | 2.995389285 | 0.002740948 |
| CDH11 | tight_junction | Up | 2.699756044 | 0.006939034 |
| CLDN5 | tight_junction | Down | 2.331602722 | 0.0197216 |
| CLDN23 | tight_junction | Up | 2.286623273 | 0.022217822 |
| CACNG4 | tight_junction | Down | 2.174416431 | 0.029673876 |
| CDH15 | tight_junction | Down | 2.171879062 | 0.029864786 |
| TJP2 | tight_junction | Down | 2.159316734 | 0.030825602 |
| CDH5 | tight_junction | Down | 2.110069291 | 0.034852388 |
| CTNNB1 | tight_junction | Down | 2.043381545 | 0.041014682 |
| CLDN11 | tight_junction | Down | 2.034241829 | 0.04192722 |
| CASK | tight_junction | Up | 1.918783621 | 0.055011724 |
| CLDN12 | tight_junction | Up | 1.809235449 | 0.070414432 |
| CLDN2 | tight_junction | Down | 1.668881275 | 0.095140912 |
| ESAM | tight_junction | Down | 1.511709988 | 0.130607656 |
| CDH18 | tight_junction | Down | 1.462971449 | 0.143475192 |
| CASKIN2 | tight_junction | Down | 1.453468581 | 0.146093696 |
| CLDN18 | tight_junction | Up | 1.416429536 | 0.156649782 |
| JAM2 | tight_junction | Down | 1.382201825 | 0.16690974 |
| CLDN19 | tight_junction | Up | 1.348941506 | 0.177355756 |
| TJAP1 | tight_junction | Down | 1.283683921 | 0.199252572 |
| SYMPK | tight_junction | Up | 1.255213422 | 0.209401294 |
| CACNG6 | tight_junction | Up | 1.227948823 | 0.21946618 |
| CTNNA2 | tight_junction | Up | 1.193575176 | 0.23264419 |
| CLDN16 | tight_junction | Up | 1.105483719 | 0.268950046 |
| CLDN9 | tight_junction | Down | 1.080492701 | 0.279922836 |
| CACNG8 | tight_junction | Down | 1.075215889 | 0.282278084 |
| CACNG3 | tight_junction | Up | 0.974508405 | 0.329804168 |
| CDH16 | tight_junction | Down | 0.793369095 | 0.427562812 |
| CDH17 | tight_junction | Down | 0.77913665 | 0.435899224 |
| CLDN20 | tight_junction | Down | 0.759537008 | 0.447531384 |
| CLDN17 | tight_junction | Down | 0.639185506 | 0.52270226 |
| CLDN14 | tight_junction | Down | 0.583273906 | 0.559708926 |
| CLDN6 | tight_junction | Up | 0.457196523 | 0.647529798 |
| CACNG2 | tight_junction | Up | 0.445408195 | 0.65602479 |
| GJA8 | tight_junction | Down | 0.26499859 | 0.791010548 |
| CLDN15 | tight_junction | Down | 0.257676134 | 0.796656866 |
| CLDN1 | tight_junction | Up | 0.18121517 | 0.856198688 |
| CASKIN1 | tight_junction | Down | 0.13702039 | 0.891014678 |
| GJA3 | tight_junction | Down | 0.115515169 | 0.908036798 |
| CTNNA3 | tight_junction | Up | 0.042131864 | 0.96639358 |
| CDH10 | tight_junction | Up | 0.016914496 | 0.986504828 |
| GJA10 | tight_junction | Up | 0.013862641 | 0.988939566 |
