# Supplementary material for: Candidate pathways and genes for prostate cancer: a meta-analysis of gene expression data
Source: BMC Med Genomics. 2009 Aug 4;2:48. doi: 10.1186/1755-8794-2-48 (PMC2731785; doi:10.1186/1755-8794-2-48)
Supplement: Additional file 10 — Changes in expression of Integrin ligands in the transition from normal prostate to localized nonmetastatic prostate cancer – NP-nMPC transition. The data provided represent changes in expression of Integrin ligands in the transition from normal prostate to localized nonmetastatic prostate cancer. [file 1755-8794-2-48-S10.doc]

Additional File 7.

**Integrin ligands differentially expressed in the transition from normal prostate to localized nonmetastatic prostate cancer — NP–nMPC transition.**

| Gene_Symbol | Z score | Direction | P_value |
| --- | --- | --- | --- |
| COL4A6 | -12.31 | Down | 8.24308E-35 |
| COL9A2 | 9.52 | Up | 2.26E-24 |
| COL17A1 | -8.89 | Down | 6.01238E-19 |
| COL6A1 | -8.50 | Down | 1.88744E-17 |
| LAMB2 | -7.32 | Down | 2.55936E-13 |
| COL14A1 | -6.63 | Down | 3.4681E-11 |
| LAMB3 | -5.95 | Down | 2.73504E-09 |
| COL4A5 | -5.94 | Down | 2.84316E-09 |
| FN1 | -5.91 | Down | 3.5164E-09 |
| LAMA4 | -5.85 | Down | 5.00934E-09 |
| LAMA2 | -5.80 | Down | 6.585E-09 |
| COL4A2 | -5.67 | Down | 1.38957E-08 |
| COL6A2 | -5.56 | Down | 2.72988E-08 |
| LAMB1 | -5.51 | Down | 3.65772E-08 |
| LAMA3 | -5.28 | Down | 1.3154E-07 |
| COL2A1 | 4.97 | Up | 6.64E-07 |
| COL28A1 | 4.96 | Up | 7.14E-07 |
| COL9A1 | -4.91 | Down | 9.18482E-07 |
| COL7A1 | -4.86 | Down | 1.16463E-06 |
| COL6A3 | -4.65 | Down | 3.31182E-06 |
| COL12A1 | 4.22 | Up | 2.4884E-05 |
| COL4A1 | -4.00 | Down | 6.42482E-05 |
| COL19A1 | 3.92 | Up | 9.037E-05 |
| COL5A3 | -3.81 | Down | 0.000140476 |
| COL8A2 | -3.39 | Down | 0.000709368 |
| COL1A2 | -3.28 | Down | 0.001023176 |
| COL21A1 | -3.08 | Down | 0.002060106 |
| COL13A1 | -3.01 | Down | 0.002578656 |
| COL4A3 | -2.68 | Down | 0.007363066 |
| COL5A1 | -2.64 | Down | 0.008404264 |
| COLQ | -2.54 | Down | 0.011227496 |
| FGG | 2.52 | Up | 0.011861866 |
| COL16A1 | -2.46 | Down | 0.013893742 |
| COL3A1 | 2.32 | Up | 0.020576354 |
| LAMA5 | -2.23 | Down | 0.025981934 |
| COL23A1 | -2.19 | Down | 0.028697948 |
| COL27A1 | -2.13 | Down | 0.033436358 |
| FGB | 2.12 | Up | 0.034398606 |
| COL1A1 | -1.71 | Down | 0.087812234 |
| COL4A3BP | 1.69 | Up | 0.090194062 |
| COL8A1 | 1.65 | Up | 0.099541368 |
| COL22A1 | -1.59 | Down | 0.112018602 |
| LAMA1 | 1.50 | Up | 0.133250536 |
| LAMC2 | 1.19 | Up | 0.233395868 |
| COL20A1 | -0.99 | Down | 0.321697556 |
| LAMB4 | 0.91 | Up | 0.363357632 |
| COL18A1 | -0.74 | Down | 0.461316124 |
| COL4A4 | -0.73 | Down | 0.46493758 |
| COL5A2 | -0.70 | Down | 0.481255268 |
| COL11A1 | 0.68 | Up | 0.499075244 |
| LAMC1 | 0.67 | Up | 0.50390388 |
| EMID2 | -0.61 | Down | 0.542334406 |
| COL9A3 | -0.60 | Down | 0.546701756 |
| COL11A2 | 0.50 | Up | 0.614965022 |
| LAMC3 | -0.43 | Down | 0.669632898 |
| COL24A1 | -0.38 | Down | 0.705527408 |
| COL6A6 | 0.31 | Up | 0.758995048 |
| COL29A1 | 0.27 | Up | 0.786973368 |
| COL25A1 | -0.26 | Down | 0.797727106 |
| FGA | 0.23 | Up | 0.816593068 |
| COL15A1 | -0.07 | Down | 0.943152958 |
